# A Remarkable Difference in Pharmacokinetics of Fluorinated Versus Iodinated Photosensitizers Derived from Chlorophyll-a and a Direct Correlation between the Tumor Uptake and Anti-Cancer Activity

**DOI:** 10.3390/molecules28093782

**Published:** 2023-04-27

**Authors:** Taur Prakash Pandurang, Joseph Cacaccio, Farukh A. Durrani, Mykhaylo Dukh, Ajyal Z. Alsaleh, Munawwar Sajjad, Francis D’Souza, Dalip Kumar, Ravindra K. Pandey

**Affiliations:** 1 Department of Chemistry, Birla Institute of Technology and Science, Pilani 333031, India; p20180447@pilani-bits-pilani.ac.in; 2 Photodynamic Therapy Center, Cell Stress Biology, Roswell Park Comprehensive Cancer Center, Buffalo, NY 14263, USA; joseph.cacaccio@roswellpark.org (J.C.); farukh.durrani@roswellpark.org (F.A.D.); dukh.mykhaylo@roswellpark.org (M.D.); 3 Department of Chemistry, University of North Texas, 1155 Union Circle, Denton, TX 76203, USA; ajyalalsaleh@my.unit.edu; 4 Department of Nuclear Medicine, University at Buffalo, The State University of New York, Buffalo, NY 14221, USA; msajjad@buffalo.edu

**Keywords:** photosensitizers, photodynamic therapy, structure-activity relationship

## Abstract

To investigate and compare the pharmacokinetic profile and anti-cancer activity of fluorinated and iodinated photosensitizers (PSs), the 3-(1′-(*o*-fluorobenzyloxy)ethyl pyropheophorbide and the corresponding meta-(*m*-) and para (*p*-) fluorinated analogs (methyl esters and carboxylic acids) were synthesized. Replacing iodine with fluorine in PSs did not make any significant difference in fluorescence and singlet oxygen (a key cytotoxic agent) production. The nature of the delivery vehicle and tumor types showed a significant difference in uptake and long-term cure by photodynamic therapy (PDT), especially in the iodinated PS. An unexpected difference in the pharmacokinetic profiles of fluorinated vs. iodinated PSs was observed. At the same imaging parameters, the fluorinated PSs showed maximal tumor uptake at 2 h post injection of the PS, whereas the iodinated PS gave the highest uptake at 24 h post injection. Among all isomers, the *m*-fluoro PS showed the best in vivo anti-cancer activity in mice bearing U87 (brain) or bladder (UMUC3) tumors. A direct correlation between the tumor uptake and PDT efficacy was observed. The higher tumor uptake of *m*-fluoro PS at two hours post injection provides a solid rationale for developing the corresponding ^18^F-agent (half-life 110 min only) for positron imaging tomography (PET) of those cancers (e.g., bladder, prostate, kidney, pancreas, and brain) where ^18^F-FDG-PET shows limitations.

## 1. Introduction

Cancer ranks as one of the leading causes of death globally. As such, it is an important barrier that prevents an increase in life expectancy [1]. When comparing groups based on race/ethnicity and sex, cancer mortality is highest in African American men and lowest in Asian/Pacific Islander women [2]. By 2040, the number of new cases per year is expected to rise to 29.5 million and the number of cancer related deaths to 16.4 million [3]. Cancer arises from the transformation of normal cells into tumor cells in a multi-stage process that generally progresses from a pre-cancerous lesion to a malignant tumor [4,5]. These changes are the result of the interaction between a person’s genetic factors and three categories of external agents, including: (a) physical carcinogens, such as ultraviolet and ionizing radiation, (b) chemical carcinogens, such as asbestos, components of tobacco smoke, aflatoxin (a food contaminant), and arsenic (a drinking water contaminant); and (c) biological carcinogens, such as infections from certain viruses, bacteria, or parasites. Cancer certainly poses a major health problem, not only due to cancer-related deaths but also because of treatment toxicities [6]. Therefore, to facilitate recovery and prevent deaths, early diagnosis and image-guided therapies are of utmost importance [7]. The common imaging techniques that are currently used for cancer are X-ray, CT scan [8], ultrasound (sonography), MRI, fluorescence, and nuclear imaging (PET/SPECT). Each modality is associated with advantages and limitations. Therefore, the development of agents with multimodality imaging capability is of utmost importance [9,10].

The most popular multimodal imaging approaches are PET and fluorescence [11]. Indeed, PET is superior considering its non-invasive, quantitative resolution of structures through deep tissue, but in contrast to PET probes which rapidly decay, fluorescence probes are stable and can also be used for image-guided surgery of certain tumors (e.g., glioblastoma). Among the PET imaging agents, ^18^F-FDG (fluorodeoxyglucose) is widely used for cancer diagnosis and staging [12]. The advantages of the ^18^F-FDG PET/CT method compared with CT and MRI are that it provides whole-body imaging maps, the viability of the tumor, and the metabolic activity of the tissue [13]. However, the ^18^F-half-life is short, and it cannot be transported long distances; therefore, it requires cyclotrons to synthesize the material at or near the imaging facility [14]. Furthermore, ^18^F-FDG is not suitable for imaging tumors of those organs where glucose metabolism is high (e.g., normal brain) [15] or for imaging bladder and kidney cancers due to its clearance from these sites, which limits its ability to differentiate between the tumor and normal organ. The development of a compound with ^124^I-isotope, a longer-life, positron-emitting isotope, has provided new opportunities in molecular imaging [16]. With a half-life of 4.2 days, the ^124^I-radionuclide can be transported a long distance from the production site and thus can also be used in those imaging facilities lacking cyclotrons.

Among heterocyclic systems, pyrrole-based compounds [17] (porphyrins, chlorins, bacteriochlorins, expanded porphyrins, phthalocyanines, porphycenes, corroles, etc.) have gained considerable interest due to their ability to localize in a variety of tumors, and they have shown great potential in fluorescence-imaging and photodynamic therapy (PDT) in cancer [18,19,20]. Some of the tetrapyrrolic systems, after appropriate modifications, have also been used for cancer imaging (fluorescence, MRI, SPECT, and PET) and PDT [21,22,23,24,25,26,27]. Among the chlorophyll-a-based analogs, the pyropheophorbides are of particular interest due to their localization and retention in tumors for a long period of time. We explored their unique characteristics in developing multifunctional agents (PET/fluorescence) with an option for cancer therapy by introducing an iodobenzyl group into the macrocycle [28]. Iodinated pyropheophorbide analog, with high fluorescence and singlet oxygen-producing ability, showed great potential in tumor imaging and photodynamic therapy in the tumors of mice. Interestingly, the corresponding ^124^I-analog (PET-ONCO), obtained by reacting the trimethyl-tin intermediate with radioactive sodium iodide (Na^124^I), showed excellent tumor imaging, even for those indications (e.g., bladder, brain, pancreas) where ^18^F-FDG-PET shows limitations [28]. Recent animal data with PET-ONCO also suggest that the formulations (e.g., Tween, Pluronic, and polyacrylamide-based nanoparticles) used as delivery vehicles also have a significant impact on the in vivo bio-distribution in various organs and tumor types [28].

One of the problems associated with FDG is its rapid pharmacokinetic profile from the system. However, unlike glucose, FDG is not reabsorbed by the renal system and is excreted through the bladder [29]. Thus, replacing ^18^F-with radionuclides of a longer half-life in deoxy-glucose will not make it a suitable candidate for PET imaging. Therefore, the objective of this study was to replace iodine with fluorine in a non-radioactive iodinated photosensitizer and investigate its pharmacokinetic profile in bladder and brain tumor models, where FDG shows limited PET-imaging abilities.

Fluorine substitution has been extensively investigated in drug research as a means of enhancing biological activity [30]. It has been shown that the introduction of a fluorine atom significantly alters the physicochemical properties of the compound due to its high electronegativity [31]. Therefore, this type of modification in a porphyrin system could have a significant impact on the biological responses of the molecule.

## 2. Results and Discussion

**Scheme 1:**
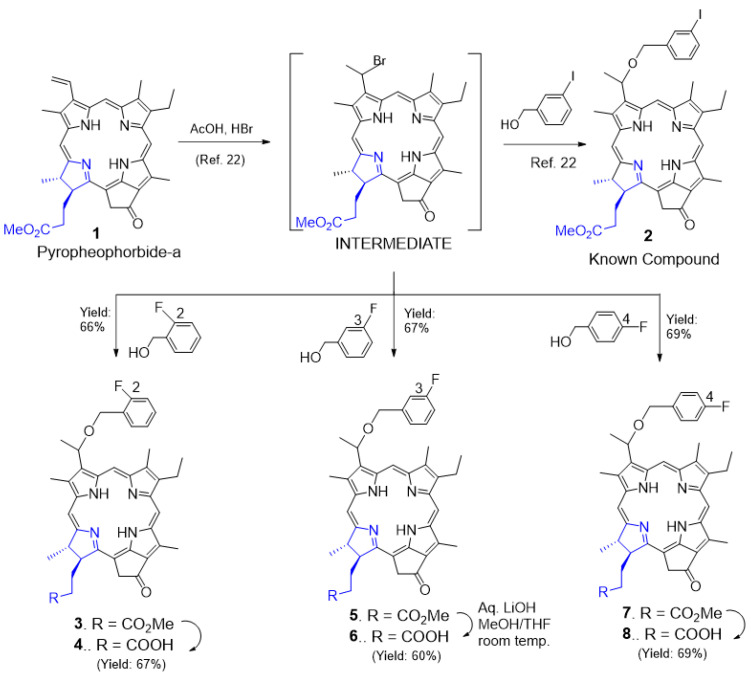
Syntheses of *o*-, *m*-, and *p*-fluoro-benzylyoxy-3-devinyl pyropheophorbides and the corresponding *m*-iodinated analog.

**Chemistry**: For the synthesis of fluorinated photosensitizer **3**, pyropheophorbide-a **1** derived from chlorophyll-a was first reacted with HBr/acetic acid. The unstable bromo-intermediate was not isolated and after drying under a vacuum was immediately reacted with *o*-*fluorobenzyl*-alcohol by following the methodology used in our laboratory (Scheme 1) for the synthesis of methyl-3-(*m*-iodobenzyloxy) ethyl-3-devinyl-pyropheophorbide-a **2**^22^. After a standard workup, the residue obtained by evaporating the solvents was dissolved in dichloromethane and treated with diazomethane to convert a small amount of the corresponding carboxylic acid (obtained by acid hydrolysis) back to methyl ester. The reaction mixture was then purified by column chromatography, and the desired methyl-3-(*o*-fluorobenzyloxy) ethyl-3-devinyl-pyropheophorbide-a **3** was obtained in a 66% yield. The same experimental procedure was followed for the preparation of *m*- and *p*-fluorobenzyloxyethyl analogs **5** and **7,** respectively. To investigate the impact of methyl ester vs. the corresponding carboxylic acids in biological efficacy, the methyl esters **3**, **5**, and **7** were separately dissolved in a mixture of methanol/THF and were reacted individually with aqueous LiOH. The PSs **4**, **6**, and **8** were isolated in moderate yields (60–69%). The structures of PSs **3**–**8** were confirmed by NMR (^1^H and ^13^C) and mass spectrometry. The purity of the photosensitizers was determined by HPLC analysis (see Supplementary Materials).

### 2.1. Photophysical Properties of Photosensitizers

**Figure 1:**
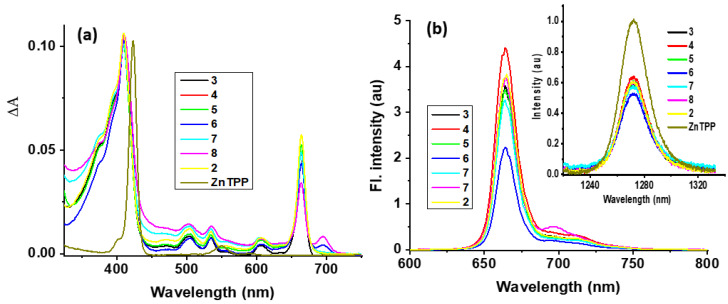
(**a**) Normalized to the Soret band absorption and (**b**) fluorescence spectrum of the indicated compounds in toluene. The samples were excited at the respective Soret peak maxima (see Table 1). The (**b**) inset shows ^1^O_2_ emissions in comparison to the tetraphenylporphyrinato zinc(II) standard.

In PDT, besides tumor specificity of a PS, its singlet oxygen producing efficiency upon exposure to light also plays a significant role in tumor destruction. The measurement of fluorescence intensity of photosensitizers also helps in determining their potential in uptake, imaging, and image-guided therapy. Therefore, the absorption and fluorescence spectral features of *o*-, *m*-, and *p*-fluorobenzyloxy derivatized pyropheophorbides **3**–**8** along with the corresponding *m*-iodinated derivative and **2** (a lead PS) were recorded. The spectra are illustrated in Figure 1, and the results are summarized in Table 1. The spectral features were very similar to the alkoxyl derivatized pyropheophorbides reported earlier [32]. A Soret band in the 410 nm region and four to five visible bands were observed. The visible band in the 665 nm region was the most intense among the visible peaks due to reduced symmetry (one reduced double bond). This trend clearly suggests a lack of interaction between the fluoro-or iodo-benzyloxy groups with the pyropheophorbide π-system. Compounds **6** and **8**, that is, -COOH derivatives of **5** and **7,** also had a less intense peak in the 695 nm region. The reasons for this are not fully clear, but it could be due to some degree of aggregation of these compounds in toluene.

All pyropheophorbide derivatives were found to be fluorescent irrespective of whether the benzyloxy groups had fluoro-or iodo-substituents. A main peak in the 664 nm range and another shoulder type peak in the 697 nm range were observed. Importantly, compound **2** with iodo-substituent was fluorescent, which suggested a lack of direct interaction with the pyropheophorbide π-system promoting heavy atom quenching [33]. The calculated fluorescence quantum yields, QY_F_, were in the 0.04–0.05 range, slightly higher than that of the ZnTPP reference of 0.033.

Next, the ability of pyropheophorbide derivatives to generate singlet oxygen (^1^O_2_) was measured. Singlet oxygen emits at ~1270 nm with a bandwidth of approximately 18 nm [34]. In the absence of direct excitation of triplet oxygen from the ground state to singlet oxygen, as this is a symmetry forbidden process, the ^1^O_2_ is often generated by chemical reactions or by photosensitization [35]. In the photosensitization process, light is absorbed by the photoactive pyropheophorbide dyes which will then transfer the absorbed energy via their triplet states to the molecular oxygen [36,37]. In the present study, we monitored singlet oxygen emission at ~1270 nm for these newly made sensitizers in O_2_-saturated toluene solution (see Figure 1b inset for spectra and the Section 4 for additional details), and the singlet oxygen quantum yields (QY_SO_) calculated as per the literature procedure are given in Table 1. The QY_SO_ values ranged between 0.43–0.50 for the investigated series of compounds without any significant effect caused by the iodo-substituent in compound **2**. In summary, the absorption and fluorescence behavior of both fluorobenzyloxy derivatives suggest a lack of interaction between the aromatic substituent with the pyropheophorbide π-system. This can also be said for fluorescence emission maxima and quantum yields; only minor changes in QY_F_ with respect to different derivatives were observed. QY_SO_ performed similarly to the moderate singlet oxygen quantum yields. These results collectively point to the ability of derivatized pyropheophorbides to retain basic absorption, fluorescence, and singlet oxygen generation properties.

### 2.2. Biological Studies

Most tetrapyrrole-based PSs are water insoluble and require a suitable delivery vehicle that is stable and non-toxic at imaging and therapeutic doses. Therefore, all PSs investigated in this study were formulated in 1% Tween 80/5% D5W and 2% (*w*/*v*) Pluronic F-127, the FDA approved formulations.

**Figure 2:**
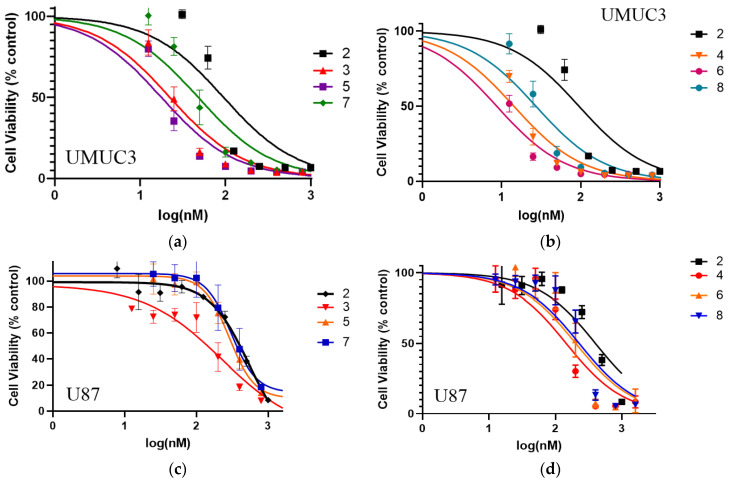
Comparative in vitro photosensitizing efficacy of PSs **2**–**8** at variable concentrations (1–3 nM) in (**a**,**b**): UMUC3 (bladder) and (**c**,**d**): U87 (brain) cell lines. The cells were incubated for 24 h before exposing them to light (1 J/cm^2^) at 665 nm. The cell viability (%) was determined by MTT assay (for details, see Section 4).

The *o*-, *m*-, and *p*-fluorinated pyropheophorbide-a analogs were synthesized to investigate the impact of the position of fluorine in benzyloxy ether side (Scheme 1) on PDT efficacy. They were also investigated for anti-cancer activity in UMUC3 (bladder) and U87 (brain) tumor cell lines via MTT assay. For an initial study, UMUC3 (bladder) cells were incubated with PSs for 24 h, the media were replaced with fresh media, and the cells were exposed to light (665 nm, 1 J/cm^2^). Cell viability was determined 24 h post PDT. Among the fluorinated photosensitizers investigated (**3**–**8**), the *o*-fluoro and *m*-fluoro derivatives (**4** and **6**, respectively) bearing a carboxylic acid functionality at position 17^2^-showed the best efficacy. They were even more effective than the iodinated analog undergoing Phase I human clinical trials (Figure 2). Interestingly, compared to U87 cells, all photosensitizers showed better efficacy (cell kill) in the UMUC3 cell line. The IC50 values (the PS dose for 50% cell kill) are summarized in Table 2.

### 2.3. Intracellular Localization of PSs in UMUC3 (Bladder) and U87 (Brain) Tumor Cells

**Figure 3:**
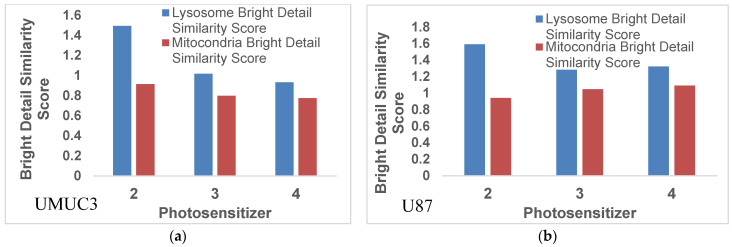
Colocalization of photosensitizer with either mitochondria or lysosomes as determined using a bright detail similarity score. The data show that PS2 preferentially localizes in the lysosomes over the mitochondria. The substitution of fluorine reduces this to almost uniform subcellular localization between lysosomes and mitochondria. This trend is cell line independent and is observed in both (**a**) UMUC3 and (**b**) U87 tumor cells.

It has been shown that the intracellular localization of a PS is also important to define the mechanism and efficiency of photoinduced cell death [38,39]. In general, most of the effective PSs localize in mitochondria and/or lysosomes, which dictate the mechanism of cell death (apoptosis vs. necrosis). To determine if the substitution of iodine with fluorine in the PS had any impact on subcellular localization (mitochondria vs. lysosome), an uptake experiment was performed in U87 and UMUC3 cell lines using the Imagestream mk II. Cells were plated in a six-well plate and allowed to adhere before adding PS, and 24 h later Lysotracker Green DND-26 and Mitotracker Red CmxRos were added to enable fluorescent labeling of the lysosome or mitochondria, respectively. The images were analyzed using Amnis IDEAS v6.2 where the bright detail similarity score was generated by comparing the site of fluorescence location of the probes in lysosomes or mitochondria to the fluorescence site specificity of the PSs. The larger the bright detail similarity score, the higher the degree of co-localization between the photosensitizer and the organelle. The results depicted in Figure 3 indicate that PS **2** (iodinated analog) showed a slight preference for lysosomes over mitochondria. Interestingly, fluorinated photosensitizers **5** (methyl ester) and **6** (carboxylic acid) decreased localization in the lysosomes without changing their localization pattern in the mitochondria, keeping the localization patterns of fluorinated and iodinated PSs similar in both U87 and UMUC3 cell lines.

**Figure 4:**
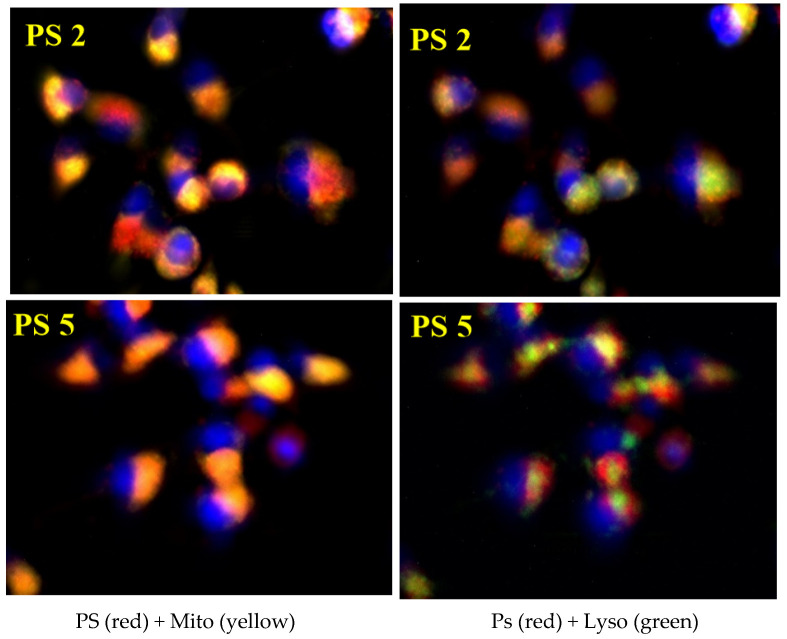
Uptake and localization of PS **2** and PS **5** in U87 cells. Cells were cultured and incubated with PS for 24 h before imaging with a Zeiss fluorescent microscope. PS fluorescence is depicted in red, lysosomes are indicated by green fluorescence, mitochondria are the yellow fluorescence, and the nucleus is blue. Both PS 531 and 201 demonstrate an almost identical sub-cellular localization pattern.

To further visualize the difference in sub-cellular localization, live cells were imaged using a Zeiss fluorescent microscope. U87 cells were incubated with either PS **2** or PS **5** for 24 and 2 h before adding Lyososensor DND 189, Mitotracker CmxRos red, and Hoeschst 33342. Both PSs **2** and **5** demonstrated a similar sub-cellular localization pattern as shown in Figure 4. Both photosensitizers showed comparable distribution throughout the cell and a significant overlap within the mitochondria. Additionally, both PSs demonstrated lysosomal localization, which was confirmed not only by the overlap of the red fluorescence from the photosensitizer with the lysotracker green fluorescence but also by the characteristic pinpoint fluorescent spots within the red fluorescence, which indicates small vessels with a high concentration of photosensitizer. In this regard, PS **2** co-localizes with the lysosomes to a higher degree than the PS **5**.

### 2.4. In Vivo Uptake and PDT Efficacy

**Figure 5:**
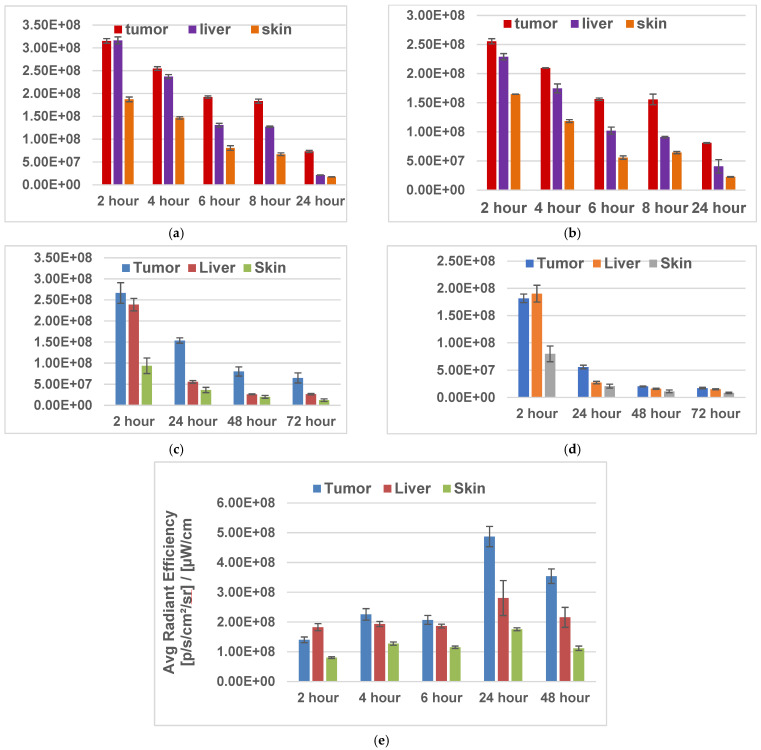
Tumor uptake of fluorinated PSs **4** and **6** and iodinated PS **2** formulated either in Tween 80 or Pluronic F-127 formulation in SCID mice (three mice/group/timepoint) bearing U87 tumors at variable timepoints. The fluorescence uptake was determined using an IVIS system. THE PSs were excited at 675 nm, and fluorescence was measured at 720 nm. [(**a**) PS **4** in Tween formulation; (**b**) PS **4** in Pluronic F-127 formulation; (**c**) PS **6** in Pluronic F-127 formulation; (**d**) PS **8** in Pluronic F-127 formulation; (**e**) PS **2** in Tween formulation].

**Figure 6:**
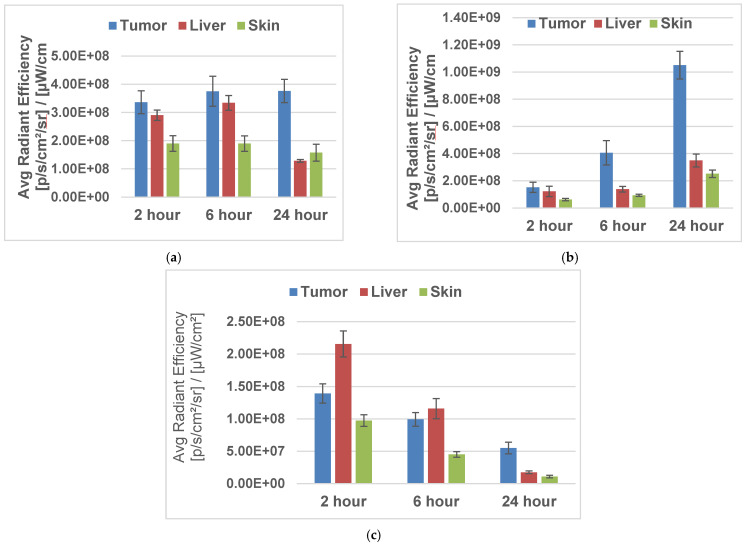
Tumor uptake of iodinated PS **2** and fluorinated PS **4** formulated either in Tween 80 or Pluronic F-127 formulation in SCID mice bearing UMUC3 tumors (three mice/group/timepoint) at variable timepoints. The fluorescence uptake was determined using an IVIS system. The PSs were excited at 675 nm, and fluorescence was measured at 720 nm. [(**a**) PS **2** in Tween formulation; (**b**) PS **2** in Pluronic F-127 formulation; (**c**) PS **4** in Pluronic F-127 formulation.

To determine the impact of delivery vehicles in tumor, liver, and skin uptake, the fluorinated PSs were formulated in Tween 80 (1%)/5% D5W and 2% (*w*/*v*) in Pluronic F-127. Initially, the in vivo biodistribution of PS **4** was undertaken at a dose of 1.0 μmole/kg at variable timepoints (2–24 h). The results depicted in Figure 4 show that both formulations provided the highest tumor uptake of PS pharmacokinetics (PK), with the highest tumor uptake at 24 h post injection instead of 2 h, as observed with the fluorinated isomers (Figure 5 and Figure 6). In SCID mice bearing U87 tumors, a parabolic relationship concerning PS **2** uptake in tumors, the liver, and skin was observed. Such a relationship was not observed in mice bearing UMUC3 tumors. The tumor and liver uptake were similar from 2–6 h, with a faster clearance from liver and skin at 24 h post injection. At all timepoints, the tumor uptake was significantly higher. However, in the same tumor model, PS **2** formulated in Tween and Pluronic F-127 formulations showed a significant difference in tumor vs. liver and skin uptake. The ortho-fluorinated PS in the Pluronic formulation showed higher uptake in tumors 2 h post injection, but the liver uptake was also significantly high at 2 h post injection and its concentration decreased slowly with time. Similar pharmacokinetic profiles were also observed in the liver and in skin but with a faster clearance rate. Replacing the formulation from Tween to Pluronic did not make a significant difference to the pharmacokinetic characteristics of the PS. The *m*- and *p*-fluorinated photosensitizers also showed similar pharmacokinetic patterns, with the highest uptake at two hours post injection. These results suggest that among the fluorinated isomers, the presence of fluorine introduced at various positions of the phenyl group in 3-(1′-benzyloxy)ethyl-3-devinylpyropheophorbide-a side chain had limited impact on their pharmacokinetic profile. Interestingly, in contrast to the fluorinated PSs, the iodinated PS **2** (*m*-iodo) showed a significant difference in optimal time of in vivo tumor uptake and clearance post injection of PSs in mice with tumors.

### 2.5. Comparative In Vivo PDT Efficacy

**Figure 7:**
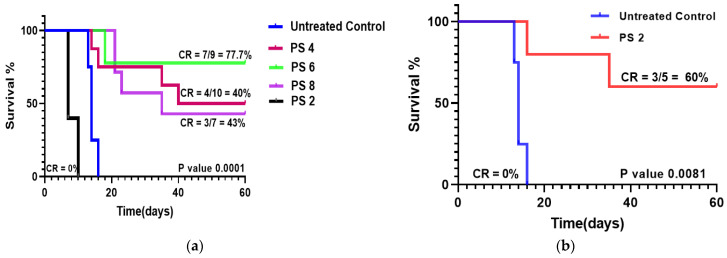
Comparative in vivo PDT efficacy of fluorinated PSs **4**, **6** and **8** and iodinated analog **2** formulated in Pluronic F-127 in SCID mice bearing U87 tumors (three mice/group). (**a**) The PSs were injected intravenously into mice with tumors at a dose of 1.0 μmole/kg. The tumors were exposed to a laser light (665 nm) at a dose of 135 J/cm^2^ and 75 mW/cm^2^ at two hours post injection, and the tumor growth was measured daily. (**b**) The mice with tumors were also injected with the same dose, and the tumors were treated with same dose of light at 24 h post injection. The tumor regrowth was measured for 60 days post injection. Among the compounds tested, PS **5** and PS**2** showed the best tumor response.

We have shown that fluorinated PSs provided optimal tumor uptake at 2 h post injection in mice bearing tumors, whereas the highest uptake with iodinated PS **2** was observed at 24 h post injection. Therefore, in order to evaluate the anti-cancer activity of these compounds, the mice injected intravenously with fluorinated PSs **4**, **5**, **6**, and **8** and iodinated PS **2**. They were exposed to light (135 J/cm^2^, 75 mW/cm^2^) at 665 nm at either 2 h or 24 h post injection (depending on the time of optimal tumor uptake of the PS), and the tumor regrowth was measured daily. The results depicted in Figure 7 indicate that the U87 tumors implanted in SCID mice when exposed to light at two hours post injection showed a significant long-term cure. Among the fluorinated derivatives, PS **6** (in which fluorine was introduced at the *meta*-position) was the most effective (seven out of nine mice were tumor-free on day 600). Interestingly, PS **2**, with low tumor uptake at two hours post injection, did not show any PDT efficacy. The mice with tumors were treated with light at similar treatment parameters (light and drug dose) used for evaluating the efficacy of PS **2**, and showed improved anti-cancer activity (60% mice were tumor-free on day 60).

**Figure 8:**
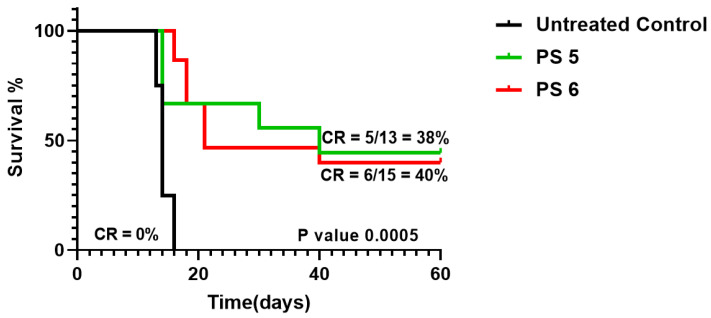
Comparative in vivo PDT efficacy of fluorinated PS **5** and **6** as methyl ester and carboxylic acid derivatives, respectively, were injected as the same drug dose (1.0 μmole/kg), and the tumors were exposed to light (135 J/cm^2^, 75 mW/cm^2^) at two hours post injection. The tumor regrowth was measured for 60 days. The PSs with methyl ester or carboxylic analogs showed similar long-term anti-cancer activity.

The photosensitizing efficacy of PS **5** bearing a methyl ester functionality at position-17^2^ was also compared with the corresponding carboxylic acid analog **6** under the same treatment parameters (drug dose 1.0 μmole/kg) in mice bearing UMUC3 tumors. The mice were exposed to light (135 J/cm^2^, 75 mW/cm^2^) at two hours post injection. The tumor growth was measured daily. Both compounds showed a similar long-term tumor cure (40% mice were tumor-free). Thus, the methyl ester vs. carboxylic acid functionality did not make any significant difference to in vivo PDT efficacy (Figure 8) This could be due to the in vivo conversion of methyl ester to carboxylic acid by the enzyme esterase.

## 3. Conclusions

Compared to iodinated PS **2**, the fluorinated PSs showed a faster uptake and clearance in mice bearing either UMUC3 (bladder) or U87 (brain) tumors. In contrast to fluorinated analogs, the iodinated PS **2** remained in circulation for a longer period and showed a significantly higher tumor uptake compared to liver and skin uptake. The maximal tumor uptake was observed at 24 h post injection. Among the fluorinated derivatives, the *m*-fluoro analog **5** treated with light at two hours post injection (the timepoint of optimal uptake in tumor in both U87 and UMUC3 tumors) showed excellent PDT efficacy (seven out of nine mice with tumors were tumor-free on day 60). The long-term tumor response (cure) results presented in this report are interesting and useful in developing tumor-specific agents for PET imaging and fluorescence-guided photodynamic therapy. Efforts are currently underway to establish an efficient approach for the synthesis of ^18^F-analog related to fluorinated PS **5** to investigate its PET imaging potential in a variety of cancer types, especially those (e.g., bladder, prostate, kidney, pancreas, and brain), where ^18^F-FDG-PET shows limitations.

## 4. Experimental Section

All the laboratory chemicals and reagents were purchased from Sigma-Aldrich, Alfa Aesar and Spectrochem India Pvt Ltd. and used as received. The reactions were monitored by thin-layer chromatography performed on Merck pre-coated plates (silica gel 60 F_254_, 0.2 mm). Column chromatographic purification of the prepared compounds was carried out using silica gel (100–200 mesh) and an ethyl acetate/hexane solvent system. Proton (^1^H), carbon (^13^C), and fluorine (^19^F) spectra were recorded on a Bruker advance II (400 MHz/100 MHz) spectrometer. Chemical shifts are reported as parts per million (δ) relative to the peak of deuterated chloroform (CDCl_3_) containing tetramethylsilane (TMS) as an internal standard. Coupling constants (*J*) are reported in hertz (Hz). An HPLC analysis was performed to determine the purity of the final prepared compounds on an Agilent 1260 Infinity II system. Mass spectra of the prepared compounds were recorded on an Agilent 6545 Q-TOF LC-MS (ESI) high-resolution mass spectrometer.

General procedure for the synthesis of methyl-3-[1′-(fluorobenzyloxy)ethyl] 3-devinyl-pyropheophorbides (**3**, **5**, and **7**): A solution of methyl pyropheophorbide-a **1** (40 mg, 0.072 mmol) in hydrobromic acid (1.5 mL, 30% solution in acetic acid) was allowed to stir at room temperature for 2 h. The excess acid was distilled off under a high vacuum, and the residue obtained was placed into dry dichloromethane (5 mL) alongside an excess amount of appropriate *fluoro*benzyl alcohol (1 mL) and anhydrous potassium carbonate (20 mg, 0.14 mmol). The reaction mixture was stirred under a nitrogen atmosphere at room temperature for 45 min. After completion of the reaction, the contents were diluted with dichloromethane (50 mL) and washed with aqueous sodium bicarbonate solution (2 × 50 mL) and then with water (200 mL). The organic layer was separated, dried over anhydrous sodium sulfate, filtered, and concentrated under reduced pressure. The syrupy residue thus obtained was chromatographed on a silica column using hexane/ethyl acetate (4: 1) as an eluent to remove excess *fluoro*benzyl alcohol, followed by elution with hexane/ethyl acetate (1: 1) to produce **3**, **5**, and **7** in good yields.

Methyl-3-[1′-(*o*-fluorobenzyloxy)ethyl]-3-devinylpyropheophorbide-a (**3**). Yield 68% (33.50 mg). Extinction coefficient value at 662 in dichloromethane: 33,497. ^1^H NMR (400 MHz, CDCl_3_) δ 9.80 (d, *J* = 2.0 Hz, 1H), 9.54 (s, 1H), 8.59 (s, 1H), 7.59−7.54 (m, 1H), 7.33 (m, 1H), 7.20−7.16 (m, 1H), 7.07 (t, *J* = 9.5 Hz, 1H), 6.11−6.06 (m, 1H), 5.30 (d, *J* = 19.8 Hz, 1H), 5.15 (d, *J* = 19.8 Hz, 1H), 4.81 (s, 2H), 4.56−4.50 (m, 1H), 4.35−4.32 (m, 1H), 3.76−3.72 (m, 2H), 3.69 (s, 3H), 3.64 (s, 3H), 3.43 (d, *J* = 2.8 Hz, 3H), 3.24 (d, *J* = 2.0 Hz, 3H), 2.74−2.68 (m, 1H), 2.63−2.55 (m, 1H), 2.37−2.28 (m, 2H), 2.19 (dd, *J* = 6.7, 2.3 Hz, 3H), 1.85 (dd, *J* = 7.3, 1.2 Hz, 3H), 1.73 (t, *J* = 7.6 Hz, 3H), −1.66 (s, 1H); ^13^C NMR (101 MHz, CDCl_3_) δ 196.13, 173.51, 171.63, 162.16, 159.71, 149.34, 144.84, 141.46, 138.85, 137.90, 130.66, 130.37, 130.33, 129.56, 129.48, 125.55, 125.54, 125.41, 124.39, 124.12, 124.09, 115.46, 115.25, 106.42, 104.14, 97.94, 97.92, 72.41, 65.00, 64.97, 51.78, 51.71, 50.11, 48.10, 30.91, 29.89, 24.67, 24.64, 23.39, 23.22, 23.19, 19.55, 17.42, 12.16, 11.26, 11.15, 11.14. ^19^F NMR (376 MHz, CDCl_3_) δ −117.72, −117.68; HRMS (ESI): *m/z* [M + H]^+^ calcd for C_41_H_43_FN_4_O_4_, 675.3347; found 675.3321; HPLC purity = 96.63%, *t*_R_ = 16.16 min.

Methyl-3-[1′-(m-fluorobenzyloxy)ethyl]-3-devinylpyropheophorbide-a (**5**). Yield 67% (32.02 mg). Extinction coefficient value at 662 in dichloromethane: 50,238. ^1^H NMR (400 MHz, CDCl_3_) δ 9.90 (s, 1H), 9.66 (s, 1H), 8.70 (s, 1H), 7.32 (d, *J* = 6.0 Hz, 1H), 7.21 (d, *J* = 11 Hz, 1H), 7.13 (d, *J* = 7.5 Hz, 1H), 7.05 (t, *J* = 6.5, 8 Hz, 1H), 6.06 (q, *J* = 10 Hz, 1H), 5.35 (d, *J* = 18.9 Hz, 1H), 5.19 (d, *J* = 20.6 Hz, 1H), 4.78 (dd, *J* = 2, 2 Hz, 1H), 4.64 (dd, *J* = 3.5, 8 Hz, 1H), 4.58 (d, *J* = 8.1 Hz, 1H), 4.39 (d, *J* = 8.1 Hz, 1H), 3.81−3.76 (m, 2H), 3.74 (s, 3H), 3.66 (s, 3H), 3.43 (d, *J* = 2.2 Hz, 3H), 3.26 (d, *J* = 1.4 Hz, 3H), 2.80−2.76 (m, 1H), 2.63−2.60 (m, 1H), 2.36−2.32 (m, 2H), 2.23 (dd, *J* = 3.0 Hz, 3.0 Hz, 3H), 1.89 (d, *J* = 7.0 Hz, 3H), 1.76 (t, *J* = 7.6 Hz, 3H), −1.70 (s, 1H); ^13^C NMR (101 MHz, CDCl_3_) δ 196.17, 173.54, 173.52, 171.51, 163.68, 161.24, 149.16, 144.99, 144.89, 141.63, 141.32, 141.30, 138.73, 137.89, 137.86, 136.27, 136.19, 135.94, 135.90, 135.52, 135.44, 134.11, 132.84, 132.79, 131.68, 130.68, 130.61, 130.53, 129.95, 129.86, 129.70, 129.19, 122.63, 115.43, 115.22, 104.15, 104.10, 104.06, 97.87, 97.16, 71.70, 70.44, 51.73, 50.08, 50.04, 48.08, 30.97, 30.91, 29.89, 29.73, 24.61, 24.57, 23.22, 23.19, 23.17, 19.53, 19.46, 17.46, 17.41, 14.36, 12.13, 12.12, 12.08, 11.33, 11.25, 11.22, 11.17, 11.15; ^19^F NMR (376 MHz, CDCl_3_) δ −113.21, −113.20, −113.19, −113.17, −113.16, −113.14; HRMS (ESI): *m*/*z* [M + H]^+^ calcd for C_41_H_43_FN_4_O_4_, 675.3347; found 675.3246; HPLC purity = 99.00%, *t*_R_ = 17.79 min.

Methyl-3-[1′-(*p*-fluorobenzyloxy)ethyl]-3-devinylpyropheophorbide-a (**7**). Yield 69% (34.09 mg). Extinction coefficient value at 662 in dichloromethane: 29,800. ^1^H NMR (400 MHz, CDCl_3_) δ 9.83 (s, 1H), 9.62 (s, 1H), 8.64 (s, 1H), 7.37−7.33 (m, 2H), 7.08−7.03 (*m*, 2H), 6.02 (q, *J* = 6 Hz, 1H), 5.32 (d, *J* = 19.9 Hz, 1H), 5.17 (d, *J* = 19.8 Hz, 1H), 4.75 (d, *J* = 11.5 Hz, 1H), 4.63 (d, *J* = 3.5 Hz, 1H), 4.55 (d, *J* = 7.1 Hz, 1H), 4.36 (d, *J* = 8 Hz, 1H), 3.79−3.75 (m, 2H), 3.72 (s, 3H), 3.65 (s, 3H), 3.41 (d, *J* = 2.8 Hz, 3H), 3.22 (d, *J* = 1.6 Hz, 3H), 2.77−2.73 (m, 1H), 2.62−2.58 (m, 1H), 2.37−2.34 (m, 2H), 2.18 (dd, *J* = 3, 4 Hz, 3H), 1.87 (d, *J* = 6.8 Hz, 3H), 1.74 (t, *J* = 7.6 Hz, 3H), −1.71 (s, 1H); ^13^C NMR (101 MHz, CDCl_3_) δ 196.12, 173.52, 173.50, 171.57, 164.27, 161.82, 149.28, 144.93, 144.86, 141.70, 141.35, 141.32, 141.08, 141.01, 138.64, 137.93, 136.36, 136.16, 135.54, 135.47, 132.91, 130.69, 130.00, 129.92, 129.19, 123.36, 123.33, 122.75, 114.83, 114.68, 114.62, 114.48, 106.41, 104.16, 104.11, 97.86, 97.20, 71.99, 70.40, 51.78, 51.72, 50.10, 50.06, 48.10, 30.97, 30.90, 29.89, 24.60, 24.57, 23.22, 23.19, 19.55, 19.50, 17.41, 12.15, 11.25, 11.22, 11.17, 11.16; ^19^F NMR (376 MHz, CDCl_3_) δ −114.96, −114.66; HRMS (ESI): *m*/*z* [M + H]^+^ calcd for C_41_H_43_FN_4_O_4_, 675.3347; found 675.3243; HPLC purity = 97.79%, *t*_R_ = 18.05 min.

General procedure for the synthesis of 3-[1′-fluorobenzyloxy)ethyl]-3-devinyl-pyro-pheophorbide acids (**4**, **6**, and **8**): To a solution of methyl-3-[1′-(fluorobenzyloxy)ethyl] 3-devinyl-pyropheo-phorbide a **3** (50 mg, 0.074 mmol) was added to THF and MeOH (3:1, 4 mL). LiOH (80 mg, 1.48 mmol) dissolved in water (4 mL) was added, and the reaction mixture was stirred at room temperature for 4 h. It was then neutralized with 2% aqueous acetic acid (5 mL) and extracted with dichloromethane (2 × 50 mL). The organic layer was washed with water (2 × 50 mL), dried over sodium sulfate, and filtered. After evaporating the solvent under a vacuum, the residue was triturated with hexane to produce **4**, **6**, and **8** in good yields.

3-[1′-(*o*-Fluorobenzyloxy)ethyl]-3-devinylpyropheophorbide-a (**4**). Yield 67% (32.8 mg). Extinction coefficient value at 662 in dichloromethane: 50,236. ^1^H NMR (400 MHz, CDCl_3_) δ 9.79 (s, 1H), 9.53 (s, 1H), 8.58 (s, 1H), 7.55 (m, 1H), 7.33−7.31 (m, 1H), 7.19−7.16 (m, 1H), 7.06 (t, *J* = 9.0 Hz, 1H), 6.07 (q, *J* = 12.2, 6.1 Hz, 1H), 5.31 (d, *J* = 19.8 Hz, 1H), 5.16 (d, *J* = 19.8 Hz, 1H), 4.80 (d, *J* = 3.2 Hz, 2H), 4.53 (dd, *J* = 12.9, 6.1 Hz, 1H), 4.36 (d, *J* = 8.5 Hz, 1H), 3.75−3.70 (m, 2H), 3.68 (s, 3H), 3.41 (d, *J* = 2.6 Hz, 3H), 3.23 (d, *J* = 2.4 Hz, 3H), 2.77−2.62 (m, 2H), 2.33 (m, 2H), 2.18 (dd, *J* = 6.6, 2.3 Hz, 3H), 1.85 (d, *J* = 7.0 Hz, 3H), 1.72 (t, *J* = 7.5 Hz, 3H), −1.65 (s, 1H); ^13^C NMR (101 MHz, CDCl_3_) δ 196.60, 171.48, 162.13, 160.38, 159.68, 155.23, 150.80, 149.00, 144.92, 141.32, 138.58, 137.69, 136.24, 135.52, 135.45, 132.81, 132.75, 130.35, 130.31, 129.50, 129.42, 128.24, 125.55, 125.41, 124.07, 124.04, 115.42, 115.21, 105.97, 104.03, 97.80, 92.74, 72.35, 64.93 64.89, 51.56, 50.02, 47.99, 29.72, 29.69, 24.57, 23.10, 23.08, 19.42, 17.40, 14.15, 11.91, 11.22, 11.06, 1.06; ^19^F NMR(376 MHz, CDCl_3_) δ −118.42, −118.33; HRMS (ESI): *m*/*z* [M + H]^+^ calcd for C_40_H_41_FN_4_O_4_, 661.3083; found 661.3185; HPLC purity = 99.32%, *t*_R_ = 12.60 min.

3-[1′-(*m*-Fluorobenzyloxy)ethyl]-3-devinylpyropheophorbide-a (**6**). Yield 60% (29.5 mg). Extinction coefficient value at 662 in dichloromethane: 42, 464. ^1^H NMR (400 MHz, CDCl_3_) δ 9.75 (s, 1H), 9.54 (s, 1H), 8.57 (s, 1H), 7.32 (dd, *J* = 2.1, 2.0 Hz, 1H), 7.21 (d, *J* = 9.3 Hz, 1H), 7.11−7.09 (m, 1H), 7.03 (t, *J* = 8.3 Hz, 1H), 6.02 (q, *J* = 6.5 Hz, 1H), 5.30 (d, *J* = 19.0 Hz, 1H), 5.16 (d, *J* = 19.8 Hz, 1H), 4.76 (dd, *J* = 2.7, 2.6 Hz, 1H), 4.62 (dd, *J* = 5.1, 5.2 Hz, 1H), 4.52 (q, *J* = 6.2 Hz, 1H), 4.35 (d, *J* = 8.2 Hz, 1H), 3.72 (d, *J* = 7.7 Hz, 2H), 3.68 (s, 3H), 3.38 (d, *J* = 2.0 Hz, 3H), 3.20 (d, *J* = 1.5 Hz, 3H), 2.75−2.63 (m, 2H), 2.36−2.30 (m, 2H), 2.19 (q, *J* = 2.9 Hz, 3H), 1.84 (d, *J* = 5.7 Hz, 3H), 1.72 (t, *J* = 7.4 Hz, 3H), −1.68 (s, 1H); ^13^C NMR (101 MHz, CDCl_3_) δ 196.49, 171.40, 160.31, 160.29, 155.23, 150.98, 149.06, 145.09, 141.24, 140.99, 138.48, 138.47, 137.87, 136.38, 135.38, 132.78, 130.45, 129.97, 129.89, 128.52, 123.36, 123.33, 114.83, 114.80, 114.67, 114.62, 114.46, 106.08, 104.22, 97.82, 92.78, 71.93, 70.36, 51.53, 50.03, 48.01, 30.54, 29.72, 29.67, 24.55, 24.52, 23.20, 19.51, 17.45, 12.10, 11.21, 11.13; ^19^F NMR (376 MHz, CDCl_3_) δ −113.17, −113.16; HRMS (ESI): *m*/*z* [M + H]^+^ calcd for C_40_H_41_FN_4_O_4_, 661.3084; found 661.3185; HPLC purity = 97.89%, *t*_R_ = 14.92 min.

3-[1′-(*p*-Fluorobenzyloxy)ethyl]-3-devinylpyropheophorbide-a (**8**). Yield 69% (33.80 mg). Extinction coefficient value at 662 in dichloromethane: 29,800. ^1^H NMR (400 MHz, CDCl_3_) δ 9.74 (s, 1H), 9.53 (s, 1H), 8.57 (s, 1H), 7.33 (bs, 2H), 7.06−7.02 (m, 2H), 6.00 (d, *J* = 5.6 Hz, 1H), 5.30 (d, *J* = 18.7 Hz, 1H), 5.15 (d, *J* = 19.2 Hz, 1H), 4.73 (d, *J* = 11.1 Hz, 1H), 4.60−4.51 (m, 2H), 4.35 (s, 1H), 3.70−3.66 (m, 5H), 3.38 (s, 3H), 3.19 (s, 3H), 2.75−2.63 (m, 2H), 2.34−2.32 (m, 2H), 2.16 (s, 3H), 1.83 (s, 3H), 1.71 (s, 3H), −1.69 (s, 1H); ^13^C NMR (101 MHz, CDCl_3_) δ 196.61, 171.55, 163.63, 161.19, 160.56, 155.08, 148.93, 144.94, 141.22, 141.18, 138.51, 138.49, 137.58, 136.15, 135.41, 135.36, 134.07, 134.05, 132.74, 132.70, 129.90, 129.82, 115.37, 115.16, 114.09, 105.96, 103.97, 97.69, 92.75, 71.62, 70.35, 51.59, 50.02, 47.98, 31.95, 29.72, 24.49, 23.04, 22.72, 19.36, 17.36, 14.15, 11.16, 11.06, 1.06; ^19^F NMR(376 MHz, CDCl_3_) δ −114.54, −114.35; HRMS (ESI): *m*/*z* [M + H]^+^ calcd for C_40_H_41_FN_4_O_4_, 661.3084; found, 661.3185; HPLC purity = 97.84%, *t*_R_ = 12.08 min.

Method for HPLC Analysis: The purity of the compounds was determined by high-performance liquid chromatography (HPLC) on an Agilent 1260 Infinity II HPLC system using a SunFire analytical column (C18, 250 mm × 4.6 mm, 5 μm) in an isocratic solvent system. The solvent system was 0.1% TFA in water and acetonitrile (5:95 *v*/*v*) with a flow rate of 1 mL/min and a run time of 20 min. The injection volume of all samples was 20 μL. Peaks were detected by UV absorption using a diode array detector between 410 to 420 nm.

Method for measuring single oxygen and fluorescence yields: The UV-visible spectral measurements were carried out with a JASCO UV-vis-near IR spectrophotometer with a Peltier temperature controller. The fluorescence and singlet oxygen emission were measured using a Spex Horiba Yvon double grating (Nanolog) UV-vis-near IR spectrometer coupled with a time-correlated, single-photon counting setup with nanoLED excitation sources (for fluorescence lifetimes). For steady-state fluorescence measurements, a photomultiplier detector was employed. For singlet oxygen emission measurements, a nitrogen-cooled InGaAs array detector was employed. A right-angle detection method was used.

Singlet oxygen emission from the chlorophyll-a-derived sensitizers was monitored at ~1270 nm using an O_2_-saturated toluene solution. The quantum yields *Φ_SO_* were calculated using the equation: *Φ_SO_* = *Φ_R_* × *I*/*I_R_* × *A_R_*/*A* × *η*^2^/*η_R_*^2^, where *Φ_SO_* and *Φ_R_* refer to the singlet oxygen quantum yield of the compound and the reference, *I* and *I_R_* refer to the area under the curve for the sample and reference, *A* and *A_R_* refer to the absorbance values of the compound and reference, and *η* is the refractive index of the solvent [40]. In the present study, tetraphenylporphyrinato zinc(II) was used as a reference with a reported *Φ_R_* value of 0.73 [41].

Method to determine in vitro PDT efficacy (MTT assay): Cells were plated in a 96-well plate between 5 × 10^4^ and 10 × 10^4^ cells per well. The cells were allowed to adhere to the plates then photosensitizer was added. The maximum dose was 1.6 μM and the minimum was 6.25 nM. The cells were incubated with the photosensitizer for 24 h then exposed to light at the appropriate wavelength. After 48 h, cell viability was read using an MTT assay and the results were examined and plotted using GraphPad Prism software.

Method to determine intracellular localization: U87 cells were plated in a 6-well plate and allowed to culture until they reached 60% confluence. A total of 5 µM of PS 531 and PS 201 were added to the growth media and incubated for 24 h. Two hours before imaging, the cells were washed with PBS and the serum-free media were replaced. A total of 5 µM of PS was added as well as 2 µM of Lysosensor Green DND 189. Twenty minutes prior to imaging, 250 nM Mitotracker CmxRos red was added. Five minutes before imaging, 1 µM of Hoechst 33342 was added. Prior to imaging, the cells were washed again with PBS and serum-free, phenol red-free media were added.

Determination of tumor uptake and in vivo PDT efficacy: The SCID mice with U87 glioma and UMUC3 urinary bladder tumors of 200–250 mm^3^ were separately injected intravenously (i.v) with photosensitizers PS **2** and PS **4** at a dose of 1 μmol/kg in 1% Tween/5% dextrose formulations. In U87 glioma, the PS **2** and PS **4** uptake in tumors were observed at 24 and 6 h post injection, respectively. In UMUC3 tumors, the PS **2** and PS **4** uptake was also observed at 24 h and 2–6 h, respectively. At this timepoint, the tumors were irradiated with light (fluence:135 J/cm^2^; fluence rate: 75 mW/cm^2^) for 30 min at 665 nm using a Lightwave™ laser diode. Mice were restrained without anesthesia in plexiglass holders designed to expose only the tumor and a 2–4 mm annular margin of skin to light. The tumor assessment and measurements were taken daily, then 3 times a week for 4 weeks, and twice a week thereafter for a total of 60 days post treatment. Tumor volume (mm^2^) was estimated using the following formula: tumor volume = ½ (L × W^2^). Two axes (mm) of the tumor (L, longest axis; W, shortest axis) were measured with the aid of a Vernier caliper. Complete tumor regression (CR) was defined as the inability to detect the tumor by palpation at the initial site of tumor appearance for more than 2 months post therapy. Partial tumor regression (PR) was defined as a ≥50% reduction in the initial tumor size. The edema, erythema, and scar formation in the treatment field was observed and recorded. Tumor regrowth for each treatment was evaluated to compare the anti-cancer efficacy.

## Figures and Tables

**Table 1:** Absorption and fluorescence peak maxima, singlet lifetime, fluorescence quantum yield (QY_F_), and singlet oxygen quantum yields (QY_SO_, see the Section 4 for measurement details) of the investigated compounds in toluene.

Compound	λ_abs_, nm	λ_em_, nm	τ_av_, ns	QY_F_	QY_SO_
**2**	410, 504, 537, 606, 664	665, 697	6.30	0.043	0.488
**3**	411, 504, 534, 605, 663	664, 697	6.89	0.043	0.466
**4**	411, 504, 534, 605, 663	664, 697	8.33	0.050	0.500
**5**	410, 503, 534, 606, 664	664, 697	6.92	0.041	0.489
**6**	410, 503, 536, 606, 664.	664, 697	6.72	0.038	0.430
**7**	410, 503, 535, 604, 664	664, 697	6.68	0.046	0.437
**8**	411, 503, 536, 604, 643	664, 697	6.68	0.046	0.487

**Table 2:** Comparative IC50 values of PSs formulated in Tween 80 formulation.

PS	IC_50_ (mM) UMUC3	IC_50_ (mM) U87	PS	IC_50_ (mM) UMUC3	IC_50_ (mM) U87
**2**	0.095	0.534	6	0.009	0.202
**3**	0.022	0.227	7	0.048	0.303
**4**	0.014	0.145	8	0.027	0.027
**5**	0.018	0.284			

## Data Availability

Contact the corresponding authors of data availability.

## Supplementary Materials

The following supporting information can be downloaded at: https://www.mdpi.com/article/10.3390/molecules28093782/s1. Molecular formula strings (CSV)^1^H, ^13^C NMR, and ^19^F spectra of new compounds **3**–**8**. HPLC chromatograms of compounds **3**–**8**.

## Sample Availability

Samples of the compounds are available from the authors.

## Abbreviations

PDT, photodynamic therapy; PS, photosensitizer; PET, positron emission tomography; FDG, fluorodeoxyglucose; HPLC, high performance liquid chromatography; IND, investigational new drug; ROS, reactive oxygen species.
