# Supplementary material for: A Remarkable Difference in Pharmacokinetics of Fluorinated Versus Iodinated Photosensitizers Derived from Chlorophyll-a and a Direct Correlation between the Tumor Uptake and Anti-Cancer Activity
Source: Molecules. 2023 Apr 27;28(9):3782. doi: 10.3390/molecules28093782 (PMC10180080; doi:10.3390/molecules28093782)
Supplement: Supplementary file 1 [file molecules-28-03782-s001.zip › molecules-2316320-supplementary.pdf]

Supporting Material Information

==

Synthesis, Photophysical and Anticancer Activity of Isomeric Fluorinated Photosensitizers with a Known Iodinated Analog Derived from Chlorophyll-a in Bladder and Brain Tumor Models: Similarities and Differences

Taur Prakash Pandurang[1], Joseph Cacaccio[2], Faruk Durrani[2], Mykhaylo Dukh[2], Ajyal Z Alsatch[3], Munawwar Sajjad[4], Francis D'Souza[3,*], Dalip Kumar[1,*], and Ravindra K Pandey[2,*]

[1] Department of Chemistry, Birla Institute of Technology and Science, Pilani, Rajasthan (India)

[2]PDT Center, Cell Stress Biology, Roswell Park Comprehensive Cancer Center, Buffalo, NY 14263 (USA)

[3]Department of Chemistry, University of North Texas, 1155 Union Circle, #305070, Denton, TX 76203-5017 (USA)

[4]Department of Nuclear Medicine, University of Buffalo, NY 14221 (USA)

LIST OF CONTENTS:

Figure S1 ^{1}H NMR spectrum of **3** Page 3

Figure S2 ^{13}C NMR spectrum of **3** Page 3

Figure S3 ^{19}F NMR spectrum of **3** Page 4

Figure S4 Mass spectrum of **3** Page 4

Figure S5 ^{1}H NMR spectrum of **5** Page 5

Figure S6 ^{13}C NMR spectrum of **5** Page 5

Figure S7 ^{19}F NMR spectrum of **5** Page 6

Figure S8 Mass spectrum of **5** Page 6

Figure S9 ^{1}H NMR spectrum of **7** Page 7

Figure S10 ^{13}C NMR Spectrum of **7** Page 7

Figure S11 ^{19}F NMR spectrum of **7** Page 8

**Figure S12**	Mass spectrum of **7**	Page 8
**Figure S13**	^{1}H NMR spectrum of **4**	Page 9
**Figure S14**	^{13}C NMR spectrum of **4**	Page 9
**Figure S15**	^{19}F NMR spectrum of **4**	Page 10
**Figure S16**	Mass spectrum of **4**	Page 10
**Figure S17**	^{1}H NMR spectrum of **6**	Page 11
**Figure S18**	^{13}C NMR spectrum of **6**	Page 11
**Figure S19**	^{19}F NMR spectrum of **6**	Page 12
**Figure S20**	Mass spectrum of **6**	Page 12
**Figure S21**	^{1}H NMR spectrum of **8**	Page 13
**Figure S22**	^{13}C NMR spectrum of **8**	Page 13
**Figure S23**	^{19}F NMR spectrum of **8**	Page 14
**Figure S24**	^{19}F NMR spectrum of **8**	Page 14
**Figure S25**	Molecular Formula Strings Spread sheet	Page 15
**Figure S26.**	HPLC analysis	Page 16
**Figure S27**	HPLC chromatogram pf **3**	Page 17
**Figure S28**	HPLC chromatogram of **5**	Page 18
**Figure S29**	HPLC chromatogram of **7**	Page 19
**Figure S30**	HPLC chromatogram pf **4**	Page 20
**Figure S31**	HPLC chromatogram of **6**	Page 21
**Figure S32**	HPLC chromatogram of **8**	Page 22

Figure S1: ^{1}H NMR spectrum of 3

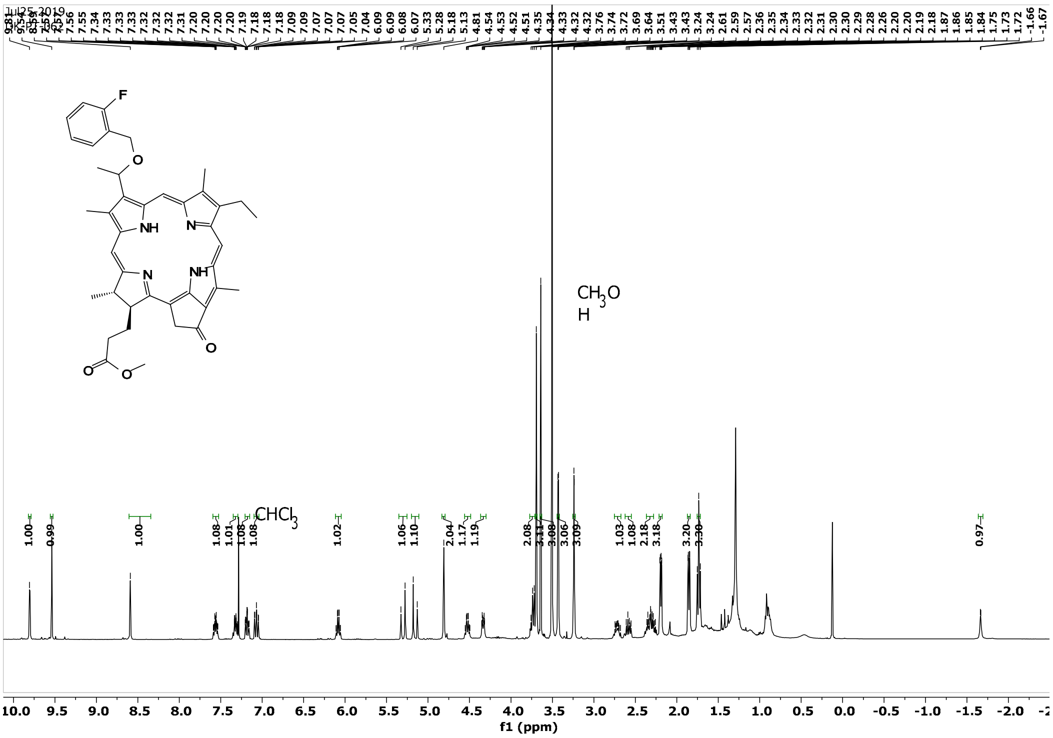

Figure S2: ^{13}C NMR spectrum of 3

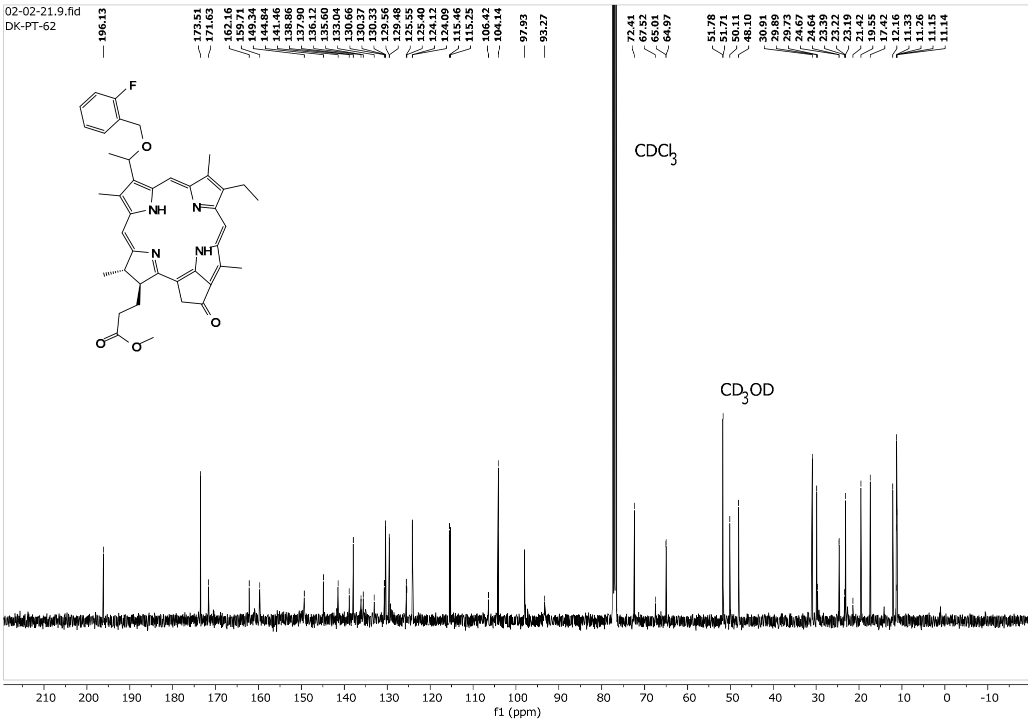

Figure S3: ^{19}F NMR spectrum of 3

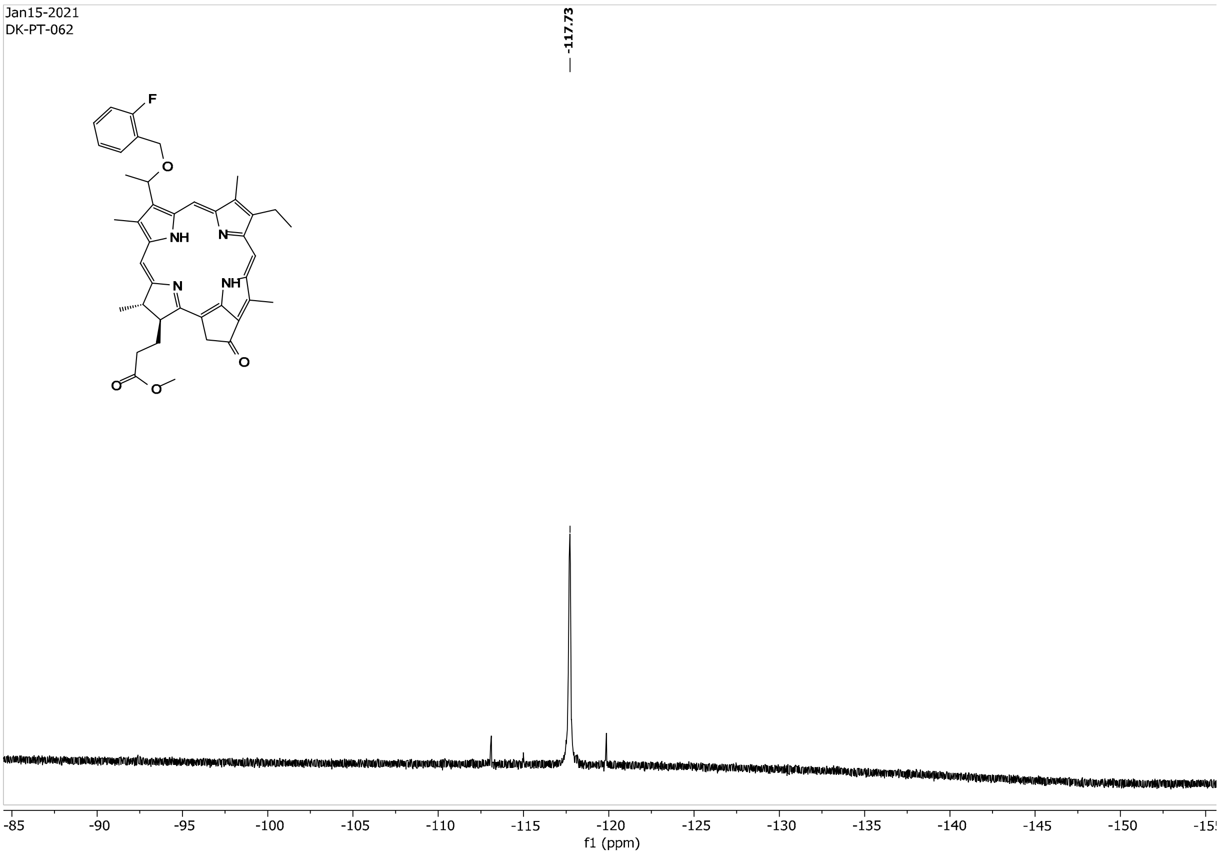

Figure S4: Mass spectrum of 3

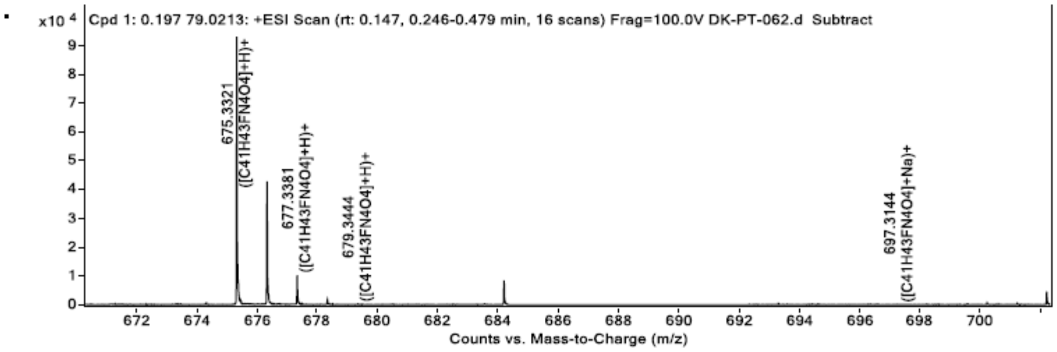

MS Spectrum Peak List

m/z	*Calc m/z*	Diff(ppm)	z	Abund	Formula	Ion
675.3321	675.3341	2.99	1	93689.75	C41H43FN4O4	(M+H)+
676.3354	676.3373	2.86	1	42870.63	C41H43FN4O4	(M+H)+
677.3381	677.3403	3.23	1	10289.9	C41H43FN4O4	(M+H)+
678.3409	678.3433	3.45	1	1688.04	C41H43FN4O4	(M+H)+
679.3444	679.3461	2.45	1	252.69	C41H43FN4O4	(M+H)+
697.3144	697.3161	2.36	1	78.07	C41H43FN4O4	(M+Na)+

Figure S5: ^{1}H NMR spectrum of 5

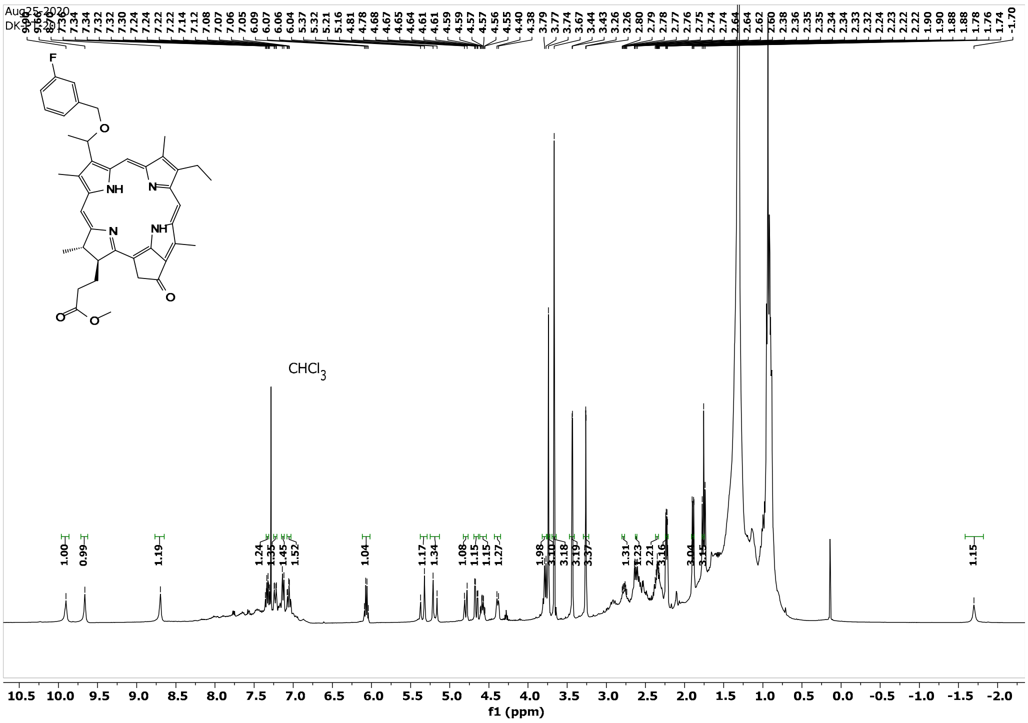

Figure S6: ^{13}C NMR spectrum of 5

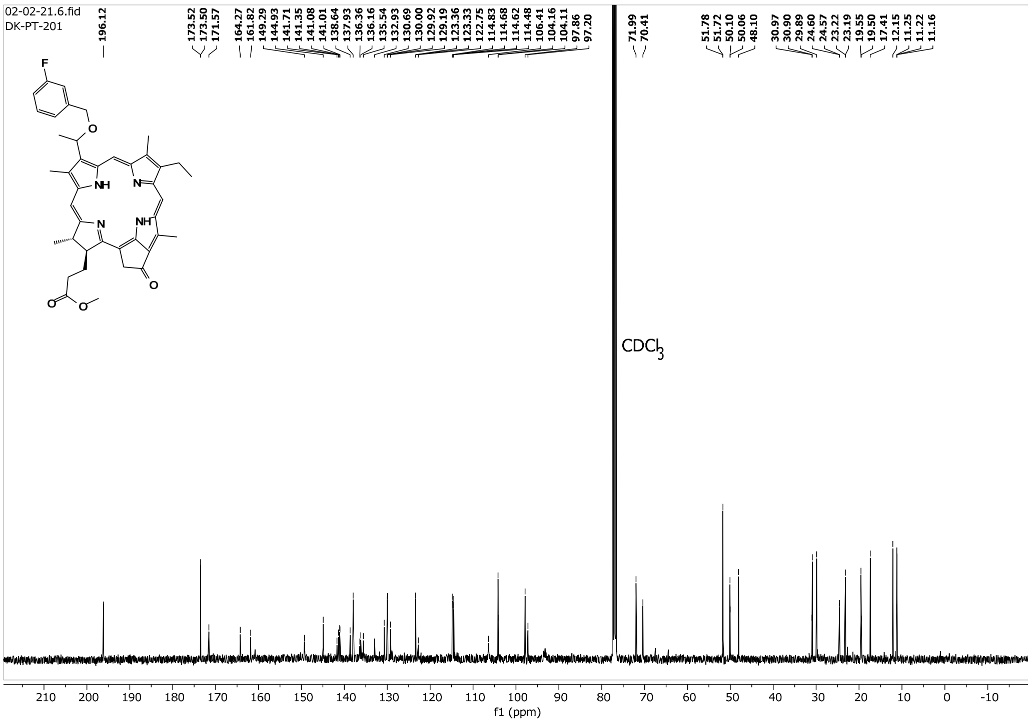

Figure S7: ^{19}F NMR spectrum of 5

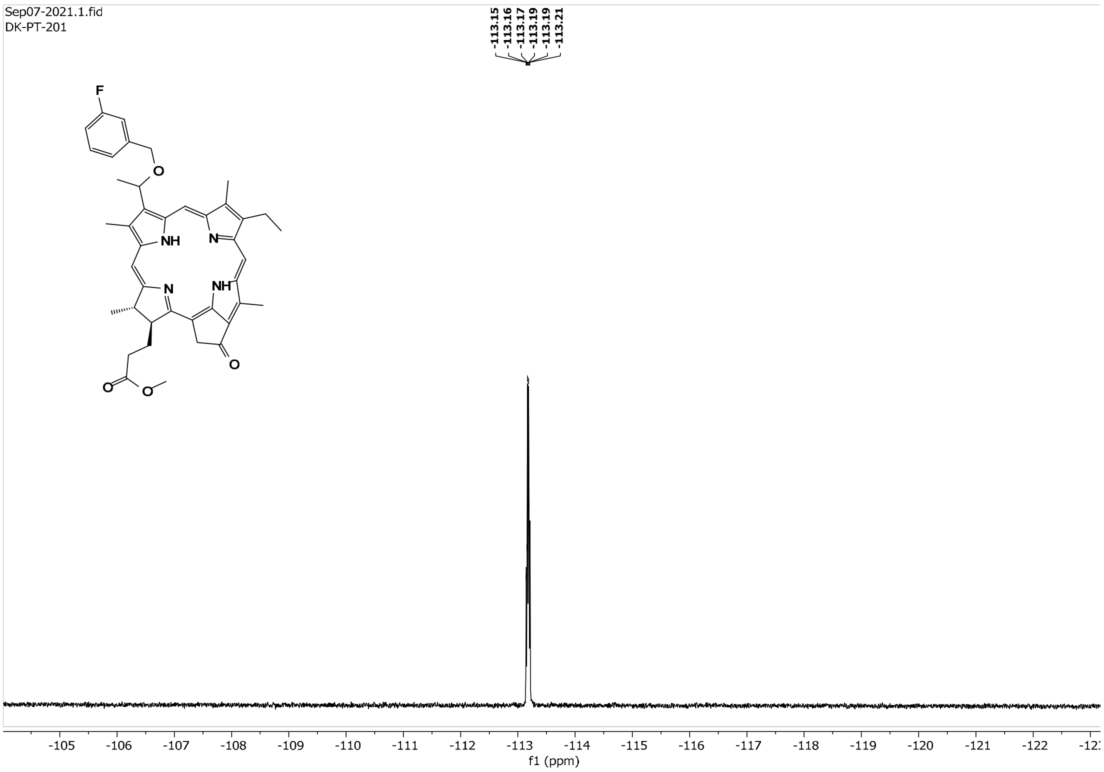

Figure S8: Mass spectrum of 5

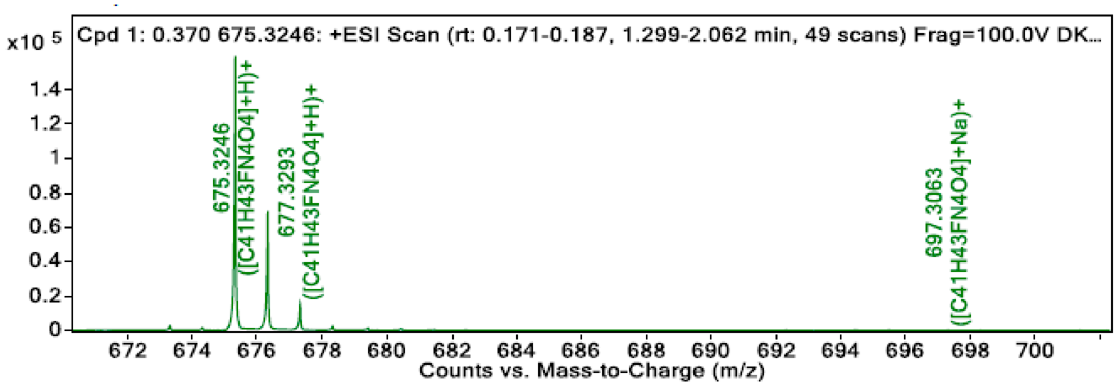

MS Spectrum Peak List

m/z	*Calc m/z*	Diff(ppm)	z	Abund	Formula	Ion
675.3246	675.3341	14.11	1	159836.96	C41H43FN4O4	(M+H)+
676.3272	676.3373	14.91	1	69399.42	C41H43FN4O4	(M+H)+
677.3293	677.3403	16.34	1	15537.04	C41H43FN4O4	(M+H)+
678.3318	678.3433	16.88	1	2574.44	C41H43FN4O4	(M+H)+
697.3063	697.3161	14.05	1	122.01	C41H43FN4O4	(M+Na)+
698.308	698.3192	16.13	1	60.22	C41H43FN4O4	(M+Na)+

Figure S9: ^{1}H NMR spectrum of 7

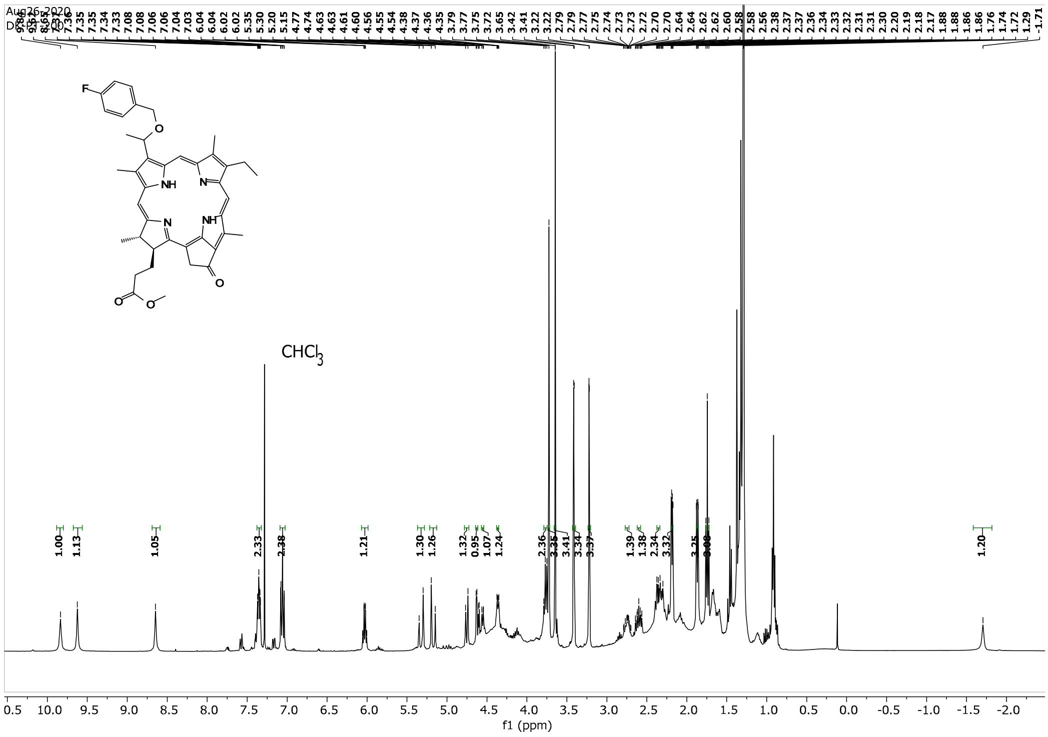

Figure S10: ^{13}C NMR spectrum of 7

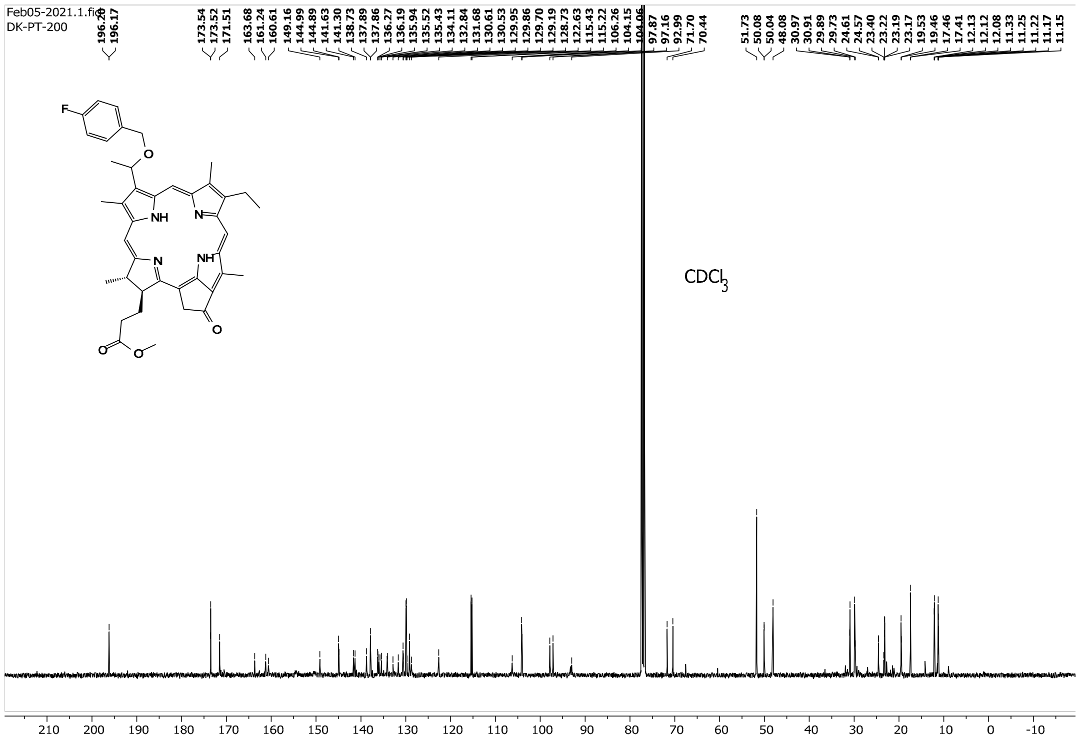

Figure S11: ^{19}F NMR spectrum of 7

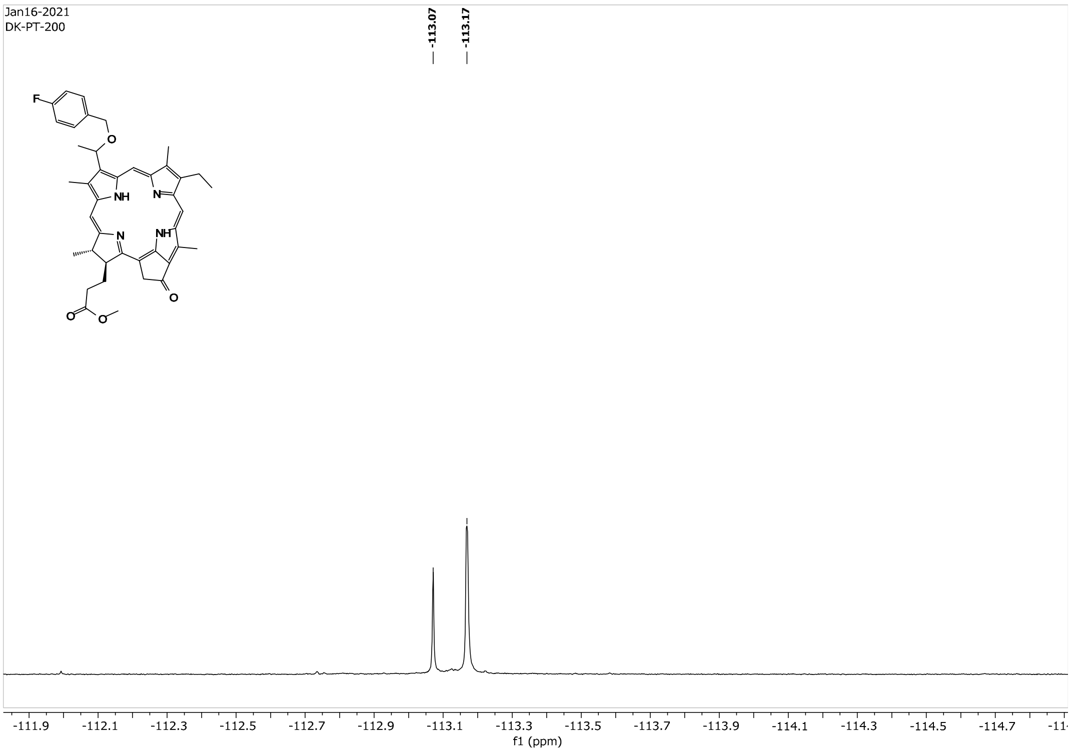

Figure S12: Mass spectrum of 7

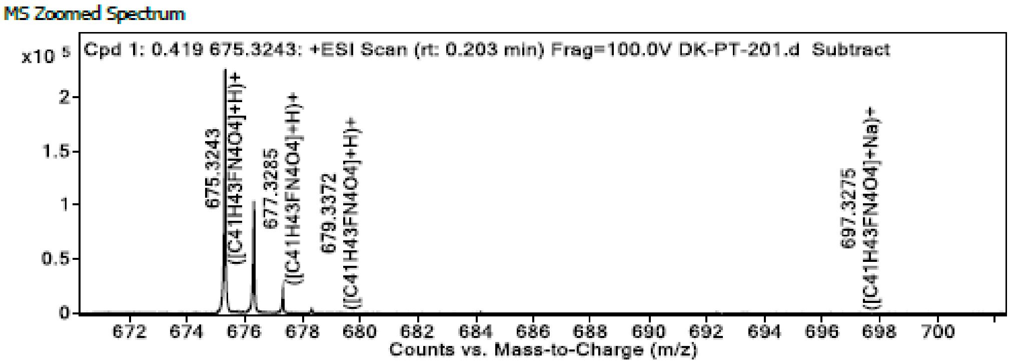

MS Spectrum Peak List

m/z	*Calc m/z*	Diff(ppm)	z	Abund	Formula	Ion
675.3243	675.3341	14.57	1	228580.18	C41H43FN4O4	(M+H)+
676.3273	676.3373	14.86	1	103767.15	C41H43FN4O4	(M+H)+
677.3285	677.3403	17.43	1	23242.15	C41H43FN4O4	(M+H)+
678.3292	678.3433	20.79	1	4192.99	C41H43FN4O4	(M+H)+
679.3372	679.3461	13.04	1	623.11	C41H43FN4O4	(M+H)+
697.3275	697.3161	-16.4	1	116.86	C41H43FN4O4	(M+Na)+

Figure S13: ^{1}H NMR spectrum of 4

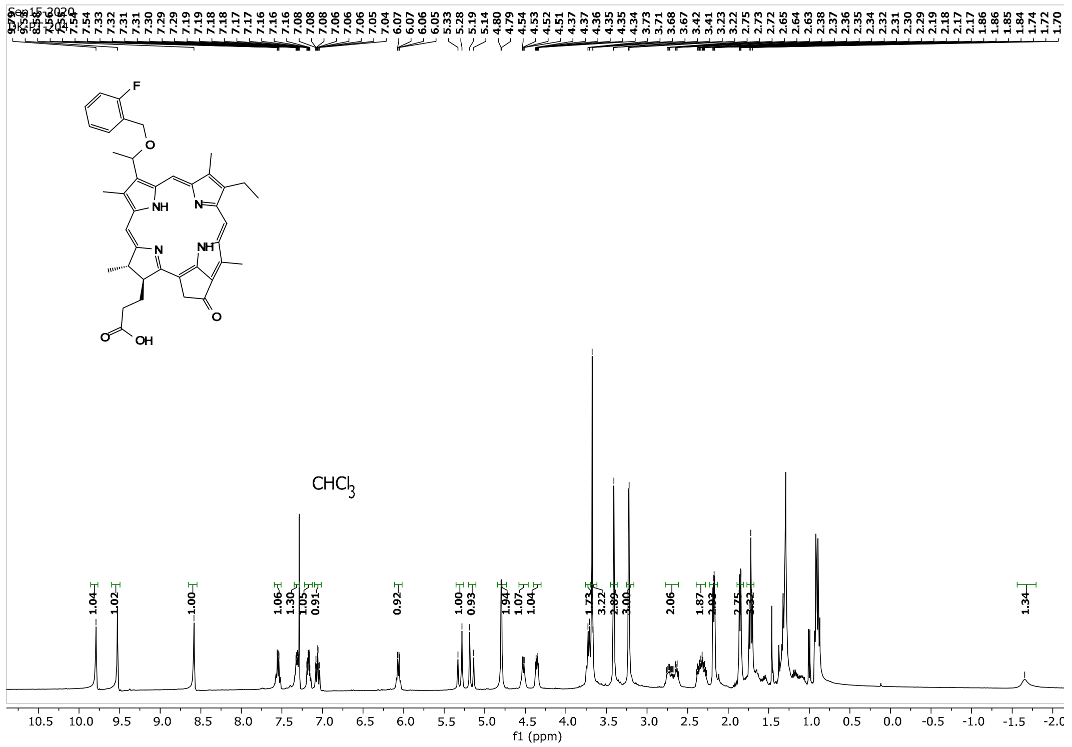

Figure S14: ^{13}C NMR spectrum of 4

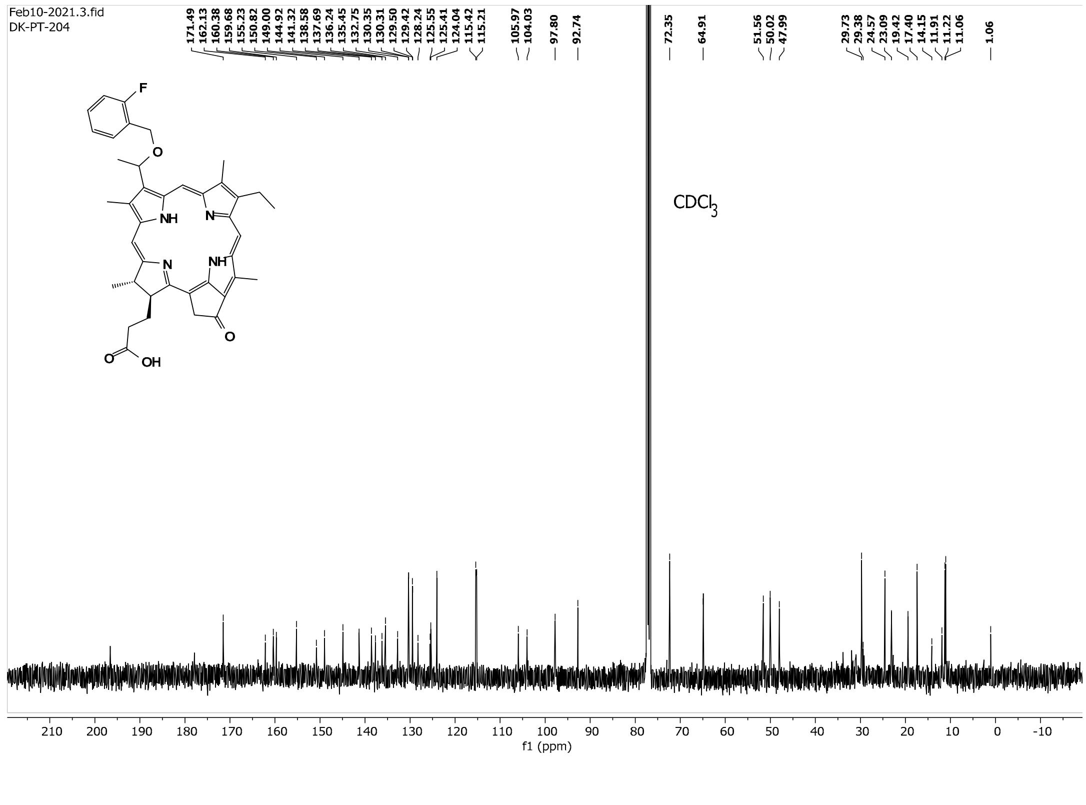

Figure S15: ^{19}F NMR spectrum of 4

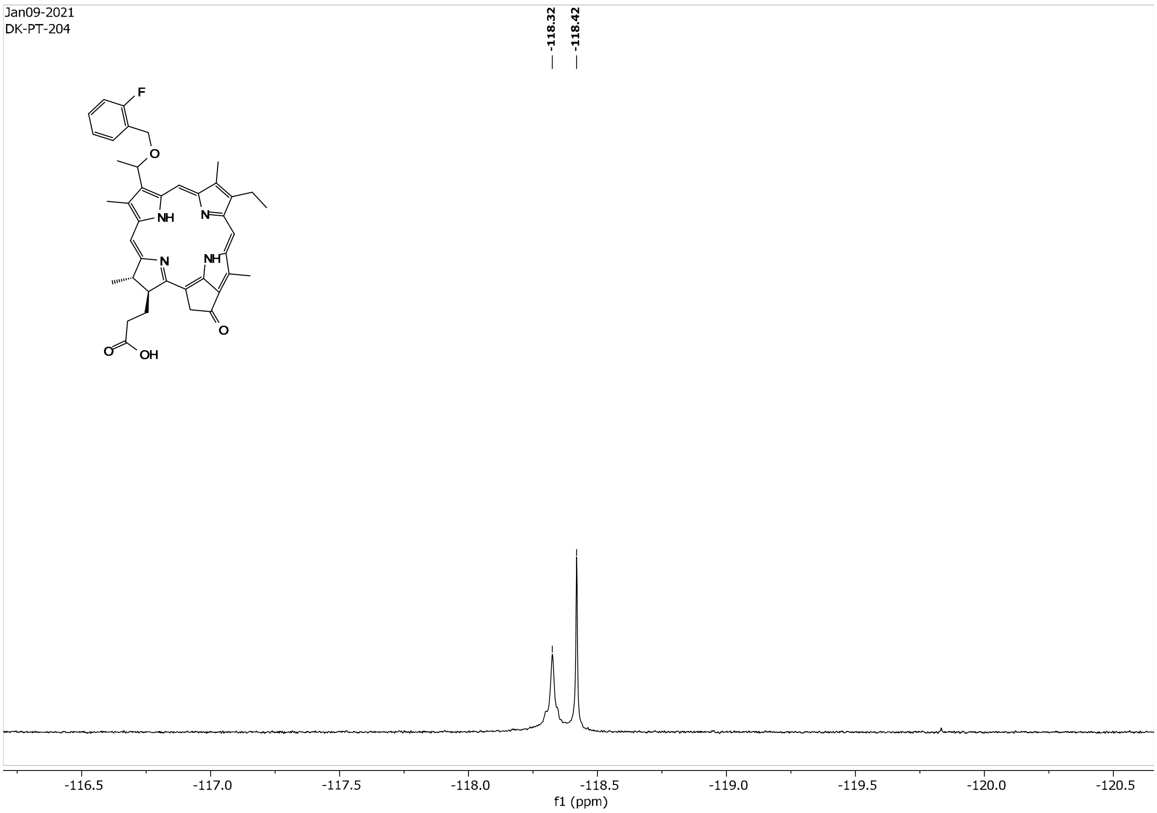

Figure S16: Mass spectrum of 4

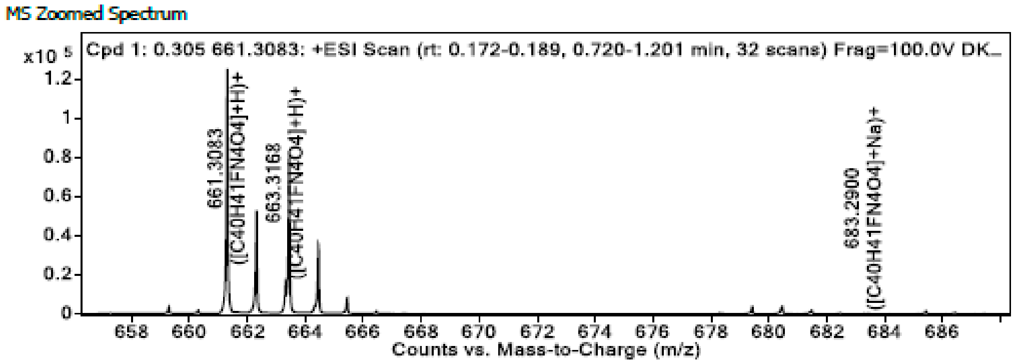

MS Spectrum Peak List

m/z	*Calc m/z*	Diff(ppm)	z	Abund	Formula	Ion
661.3083	661.3185	15.3	1	125380.18	C40H41FN4O4	(M+H)+
662.3111	662.3216	15.95	1	53149.38	C40H41FN4O4	(M+H)+
663.3168	663.3247	11.93	1	15932.4	C40H41FN4O4	(M+H)+
683.29	683.3004	15.17	1	121.78	C40H41FN4O4	(M+Na)+
684.2936	684.3036	14.66	1	63.1	C40H41FN4O4	(M+Na)+

Figure S17: ^{1}H NMR spectrum of 6

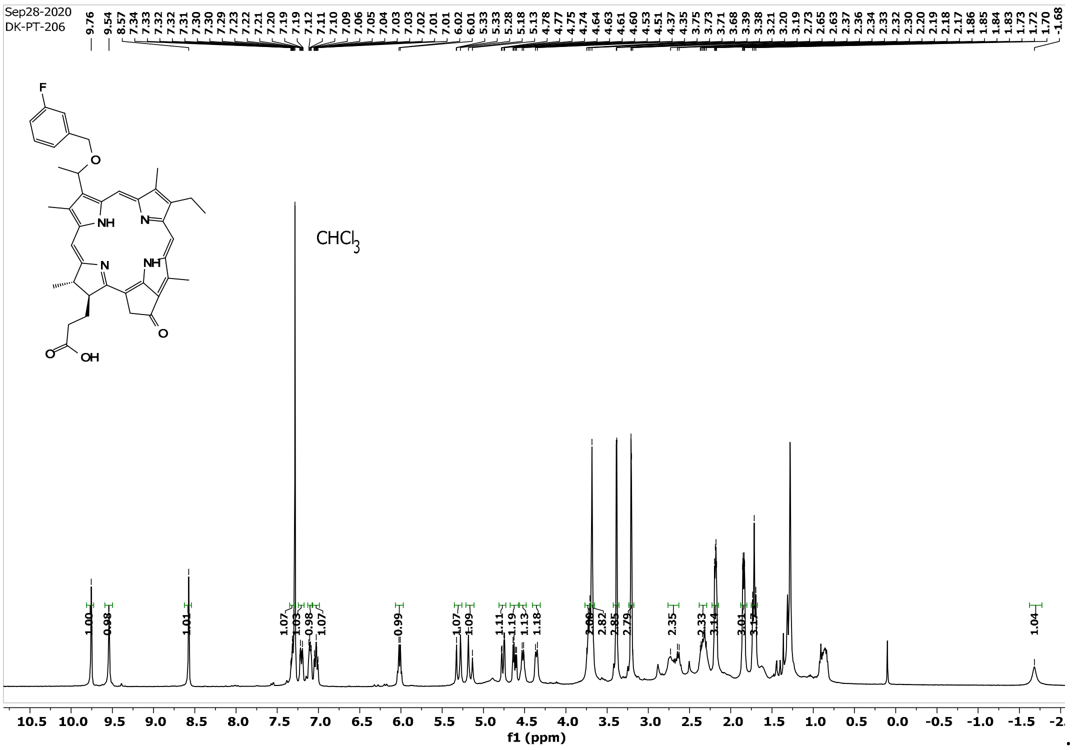

Figure S18: ^{13}C NMR spectrum of 6

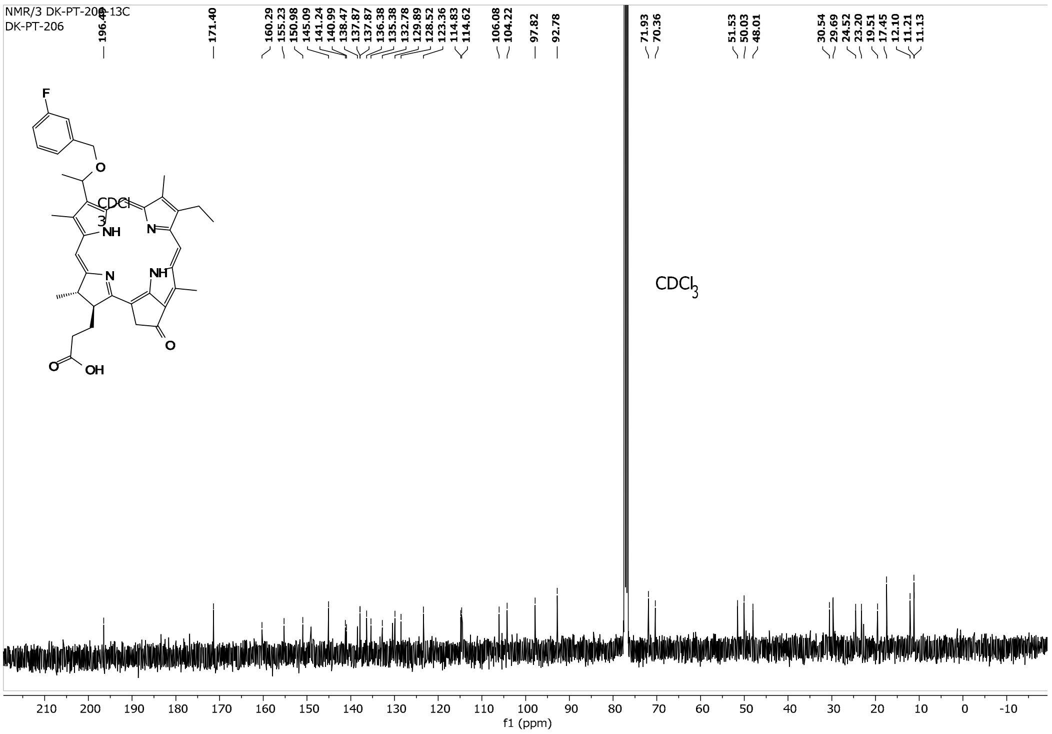

Figure S19: ^{19}F NMR spectrum of 6

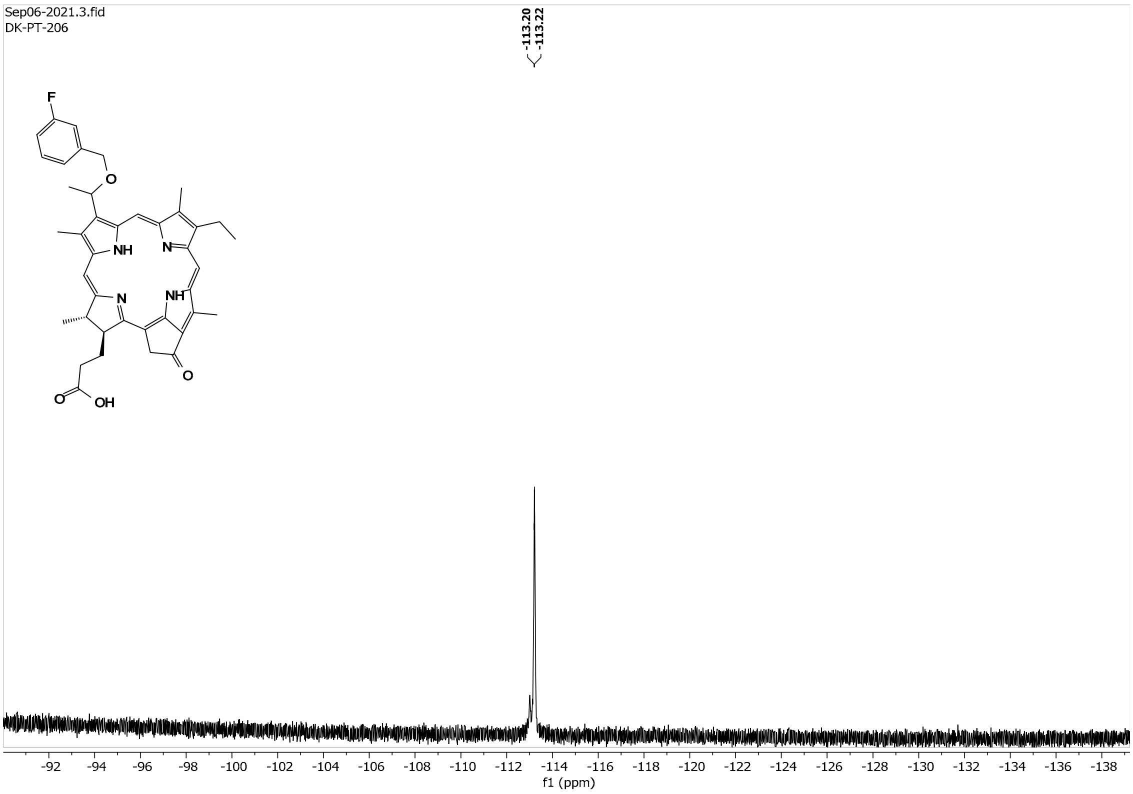

Figure S20: Mass spectrum of 6

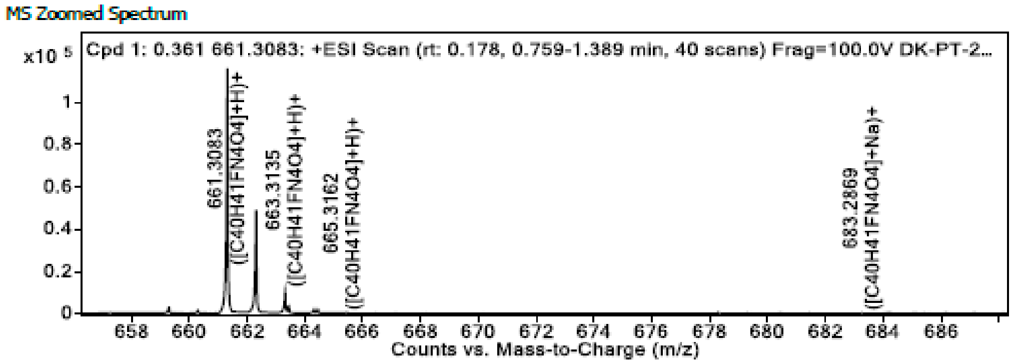

MS Spectrum Peak List

m/z	*Calc m/z*	Diff(ppm)	z	Abund	Formula	Ion
661.3083	661.3185	15.39	1	116156.56	C40H41FN4O4	(M+H)+
662.311	662.3216	16.15	1	49524.24	C40H41FN4O4	(M+H)+
663.3135	663.3247	16.76	1	11306.08	C40H41FN4O4	(M+H)+
664.3166	664.3276	16.58	1	1973.33	C40H41FN4O4	(M+H)+
665.3162	665.3304	21.37	1	240.83	C40H41FN4O4	(M+H)+
683.2869	683.3004	19.82	1	142.26	C40H41FN4O4	(M+Na)+
684.295	684.3036	12.57	1	66.94	C40H41FN4O4	(M+Na)+

Figure S21: ^{1}H NMR spectrum of 8

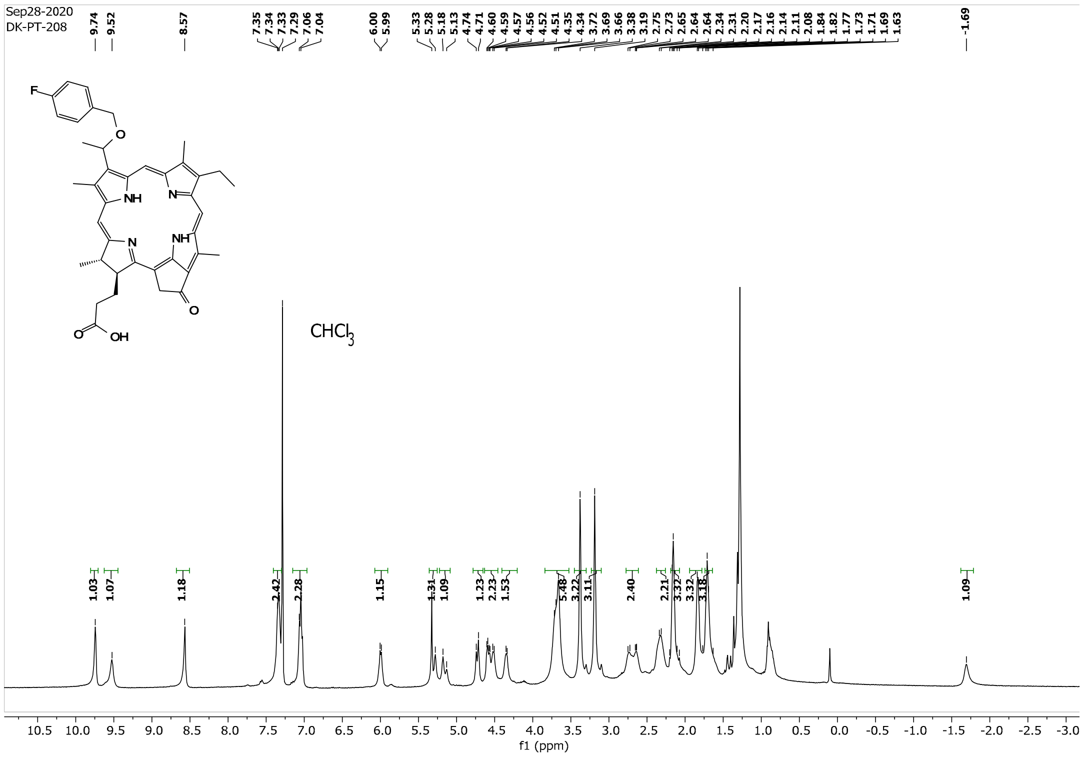

Figure S22: ^{13}C NMR spectrum of 8

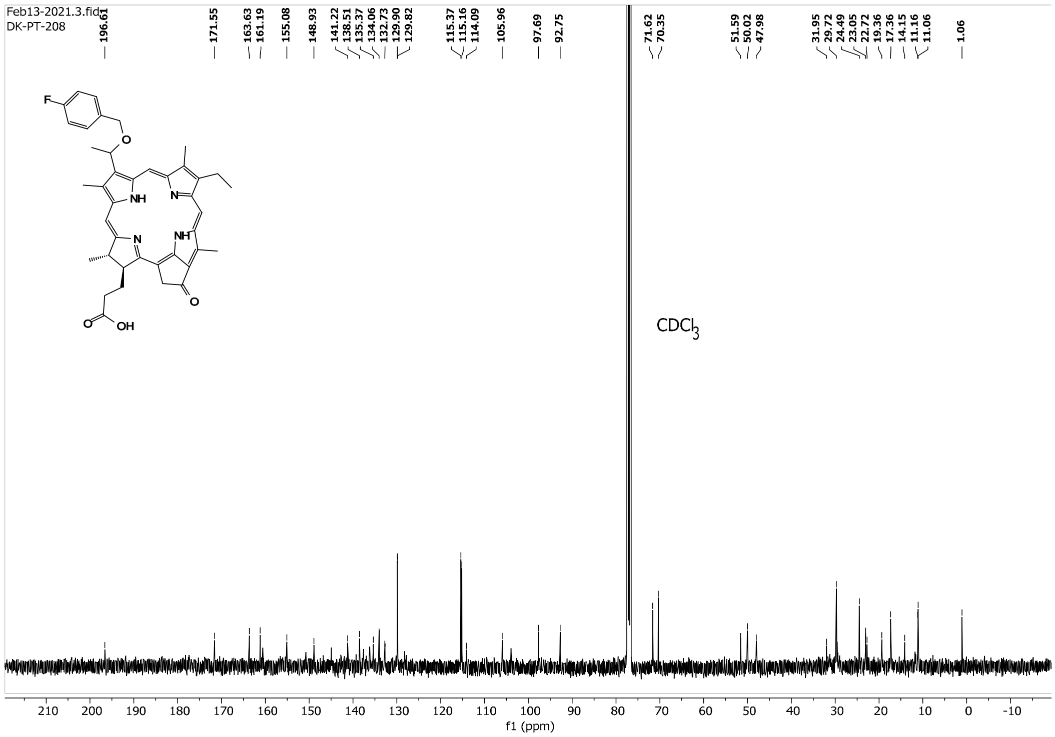

Figure S23: ^{19}F NMR spectrum of 8

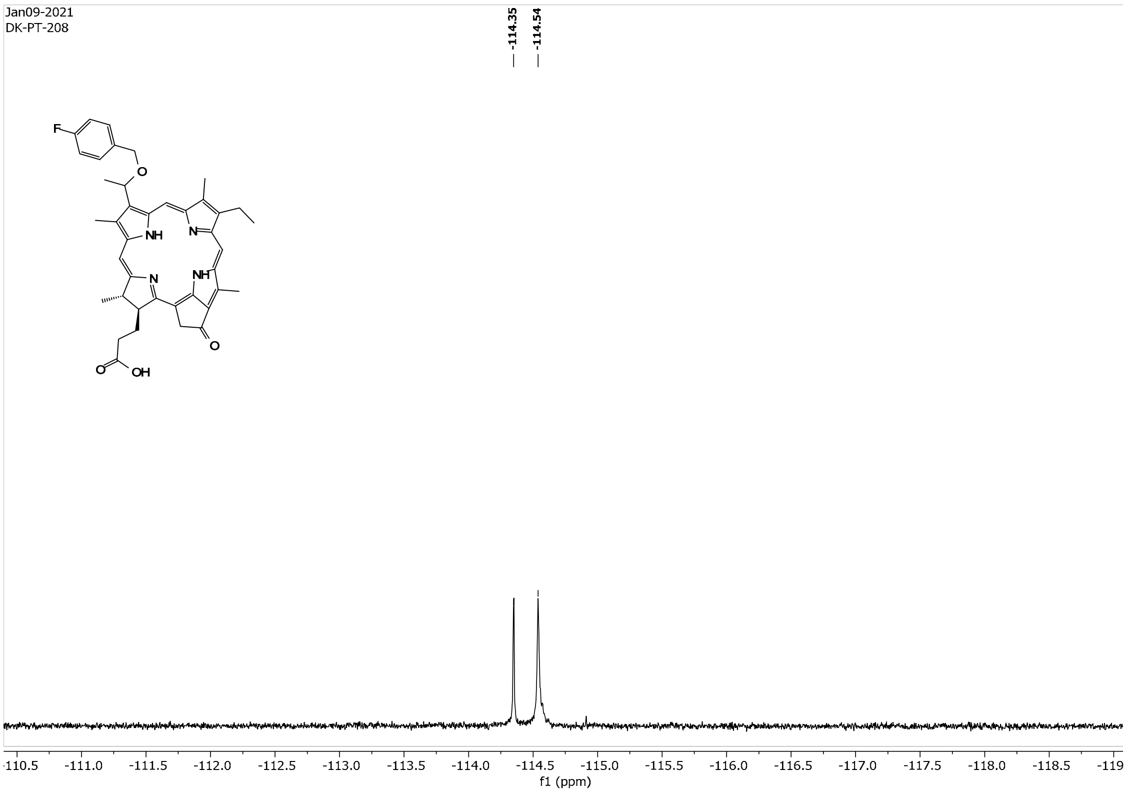

Figure S24: Mass spectrum of 8

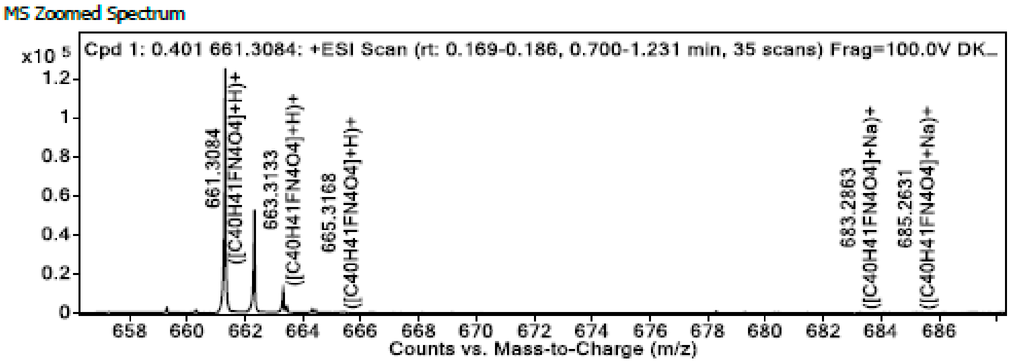

MS Spectrum Peak List

m/z	*Calc m/z*	Diff(ppm)	z	Abund	Formula	Ion
661.3084	661.3185	15.18	1	125126.84	C40H41FN4O4	(M+H)+
662.3109	662.3216	16.18	1	53093.64	C40H41FN4O4	(M+H)+
663.3133	663.3247	17.13	1	12100.66	C40H41FN4O4	(M+H)+
664.3159	664.3276	17.64	1	2109.61	C40H41FN4O4	(M+H)+
665.3168	665.3304	20.46	1	342.14	C40H41FN4O4	(M+H)+
683.2863	683.3004	20.69	1	186.88	C40H41FN4O4	(M+Na)+
684.2862	684.3036	25.47	1	88.82	C40H41FN4O4	(M+Na)+
685.2631	685.3066	63.51	1	60.77	C40H41FN4O4	(M+Na)+

Figure S25: Molecular Formula Strings Spread sheet

Compound	**Molecular Formula Strings**
**3a**	O=C1CC(C2=N/C([C@@H](C)[C@@H]2CCC(OC)=O)=C\3)=C(C1=C/4C)NC4=C/C(C(CC)=C/5C)=NC5=C/C6=C(C(OCC7=CC=CC=C7F)C)C(C)=C3N6
**3b**	O=C1CC(C2=N/C([C@@H](C)[C@@H]2CCC(OC)=O)=C\3)=C(C1=C/4C)NC4=C/C(C(CC)=C/5C)=NC5=C/C6=C(C(OCC7=CC=CC(F)=C7)C)C(C)=C3N6
**3c**	O=C1CC(C2=N/C([C@@H](C)[C@@H]2CCC(OC)=O)=C\3)=C(C1=C/4C)NC4=C/C(C(CC)=C/5C)=NC5=C/C6=C(C(OCC7=CC=C(F)C=C7)C)C(C)=C3N6
**4a**	O=C1CC(C2=N/C([C@@H](C)[C@@H]2CCC(O)=O)=C\3)=C(C1=C/4C)NC4=C/C(C(CC)=C/5C)=NC5=C/C6=C(C(OCC7=CC=CC=C7F)C)C(C)=C3N6
**4b**	O=C1CC(C2=N/C([C@@H](C)[C@@H]2CCC(O)=O)=C\3)=C(C1=C/4C)NC4=C/C(C(CC)=C/5C)=NC5=C/C6=C(C(OCC7=CC=CC(F)=C7)C)C(C)=C3N6
**4c**	O=C1CC(C2=N/C([C@@H](C)[C@@H]2CCC(O)=O)=C\3)=C(C1=C/4C)NC4=C/C(C(CC)=C/5C)=NC5=C/C6=C(C(OCC7=CC=C(F)C=C7)C)C(C)=C3N6

Figure S26: HPLC Analysis

Method(s) for HPLC analysis:

A. Instrument: Agilent 1260 Infinity II system with diode array detector

B. HPLC parameter: Solvent flow rate: 1.0 mL/min Mobile phase 0.1% (v/v) TFA in water/acetonitrile (5%) over 20 min, Data collected between 410 nm to 420 nm.

C. HPLC Column: Sunfire C18 analytical column (5 µm 4.6 $\times$ 250 mm)

Compound	Retention times (min)	Purity (%)
**3**	16.163	96.63
**5**	17.799	99.01
**7**	18.059	97.79
**4**	12.605	99.32
**6**	14.927	97.89
**8**	12.081	97.84

Figure S27: HPLC chromatogram of 3

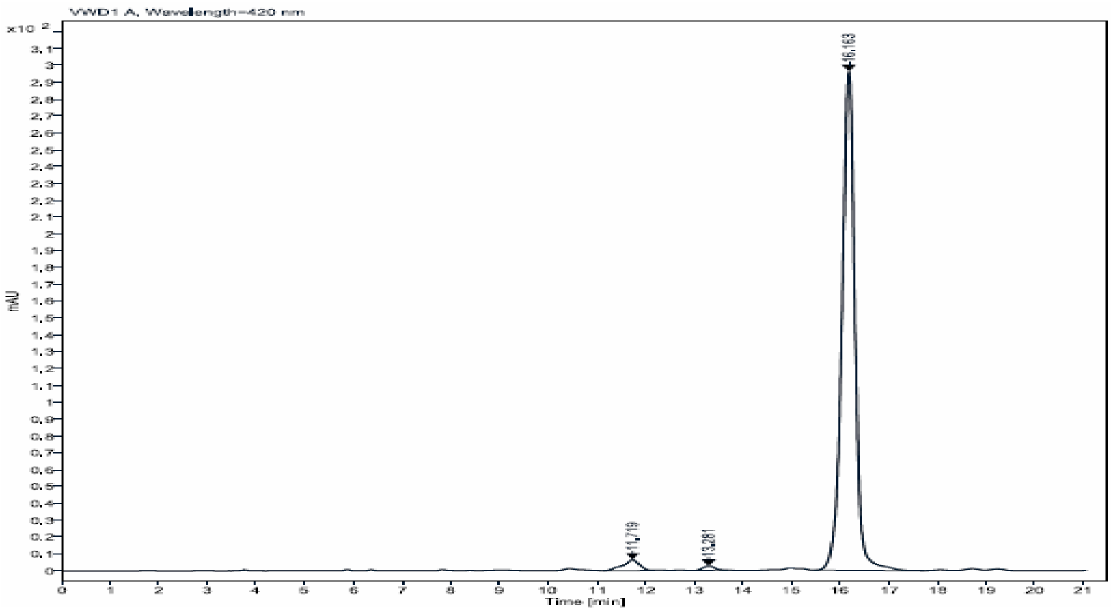

RT [min]	Type	Width [min]	Area	Height	Area%	Name
11,719	BB	0,3410	147,5701	6,2381	2,5915	
13,281	BB	0,2731	43,7973	2,5533	0,7691	
16,163	BB	0,2827	5503,0796	296,2810	96,6394	
		Sum	5694,4471			

Figure S28: HPLC chromatogram of 5

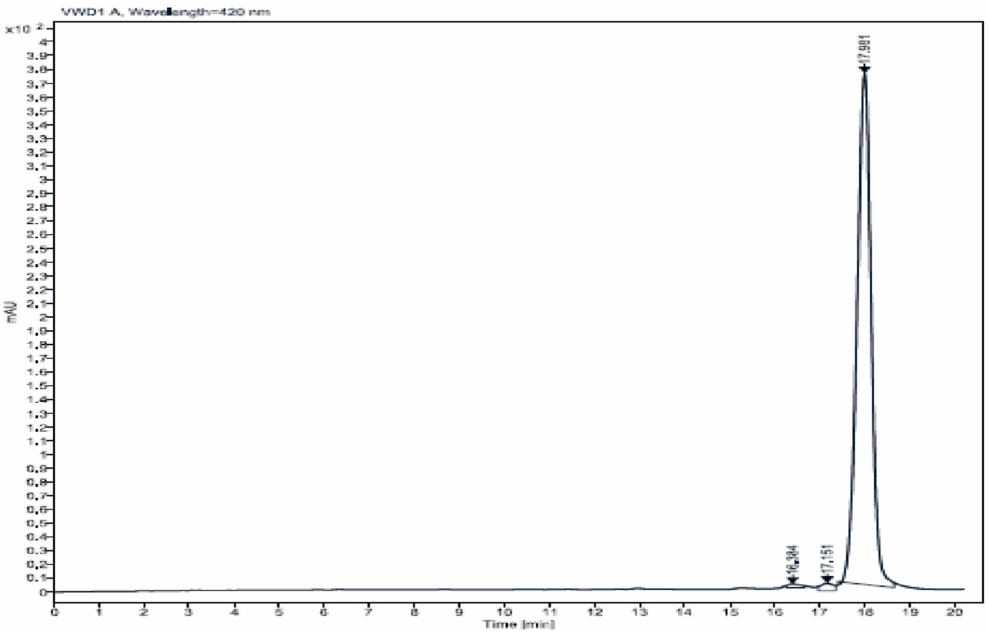

RT [min]	Type	Width [min]	Area	Height	Area%	Name
16.384	MM	0.3623	52.4034	2.4106	0.6307	
17.151	MM	0.3239	102.7382	5.2870	1.2365	
17.981	MM	0.3656	8153.4653	371.7141	98.1328	
		Sum	8308.6069			

Figure S29. HPLC chromatogram of 7

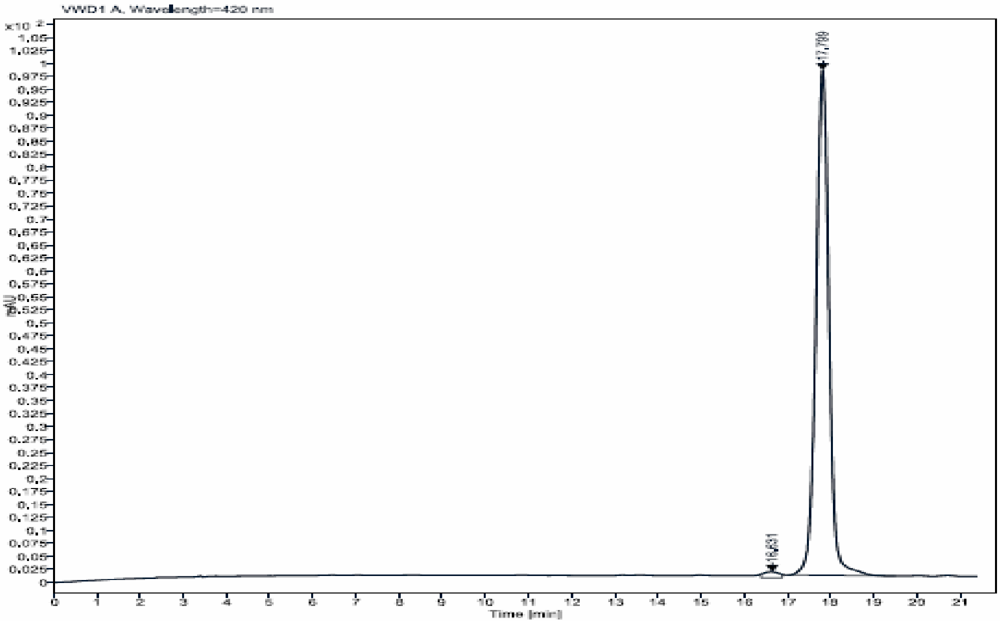

RT [min]	Type	Width [min]	Area	Height	Area%	Name
16,631	MM	0,4132	28,1894	1,1369	1,3657	
17.799	BB	0.3198	2035.9336	97.4094	98.6343	
		Sum	2064.1230			

Figure S30. HPLC chromatogram of 4

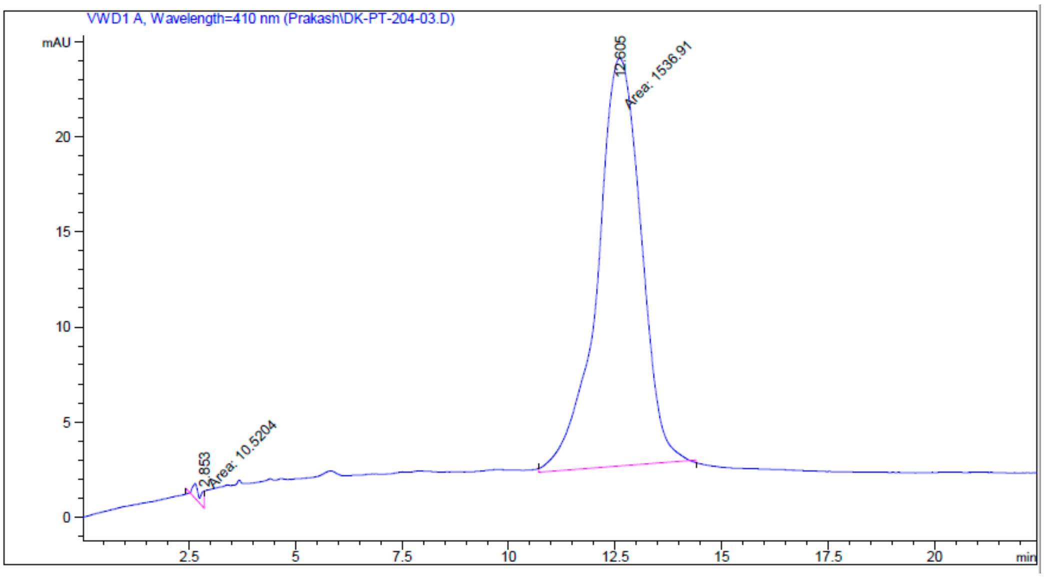

Signal 1: VWD1 A, Wavelength=410 nm

Peak #	RetTime [min]	Type	Width [min]	Area [mAU*s]	Height [mAU]	Area %
1	2.853	MM	0.1966	10.52040	8.91656e-1	0.6799
2	12.605	MM	1.1947	1536.90894	21.44054	99.3201

Figure S31. HPLC chromatogram of 6

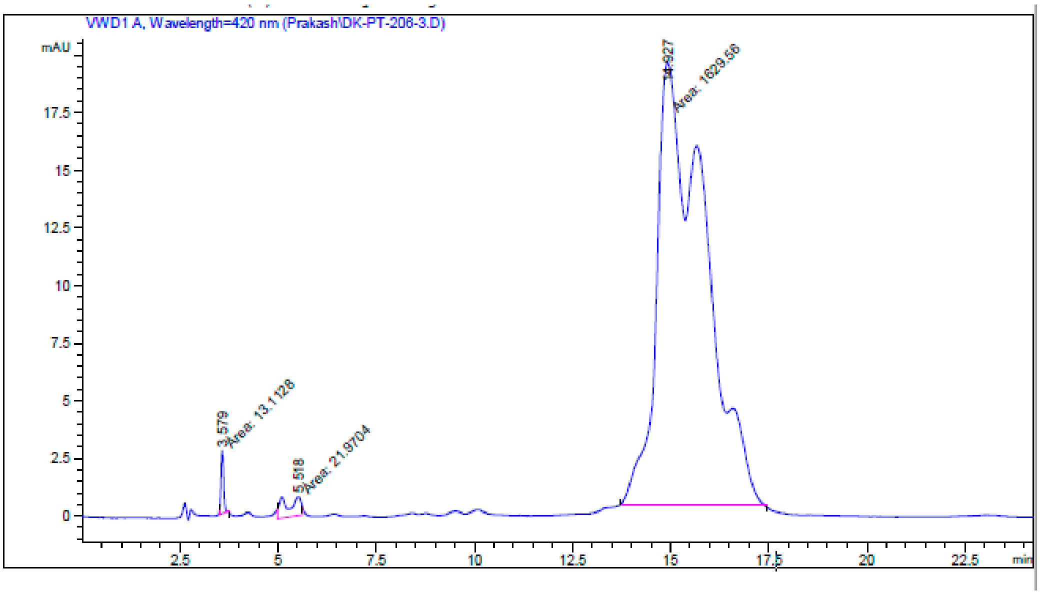

Peak #	RetTime [min]	Type	Width [min]	Area [mAU*s]	Height [mAU]	Area %
1	3.579	MM	0.0800	13.11280	2.73324	0.7877
2	5.518	MM	0.4536	21.97040	8.07332e-1	1.3198

Figure S32: HPLC chromatogram of 8

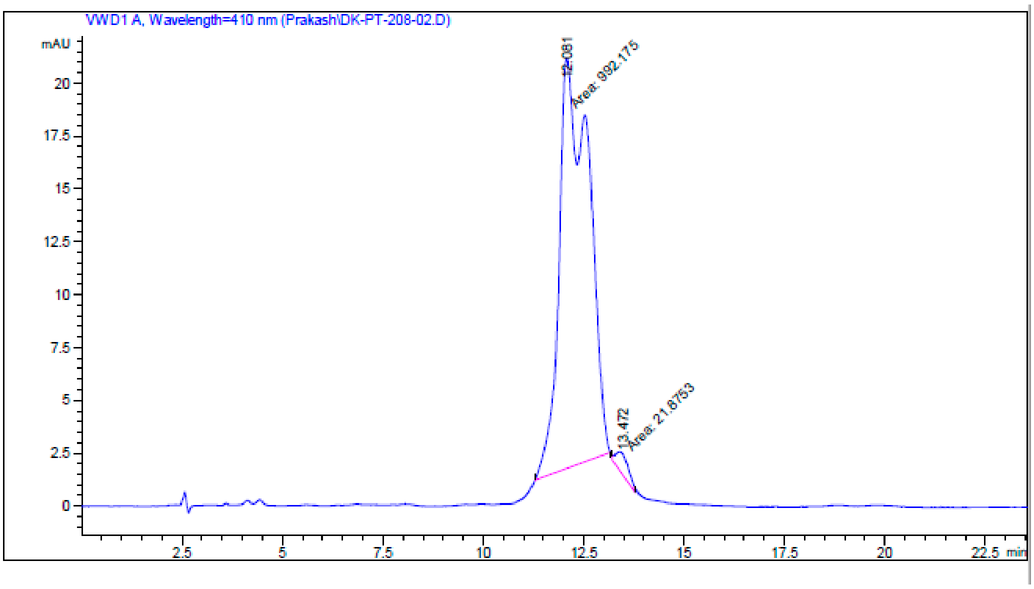

Peak #	RetTime [min]	Type	Width [min]	Area [mAU*s]	Height [mAU]	Area %
1	12.081	MM	0.8530	992.17529	19.38589	97.8428
2	13.472	MM	0.3649	21.87533	9.99117e-1	2.1572
